# An algorithm for equilibrium problems with mixed-type fixed point constraints

**DOI:** 10.1371/journal.pone.0318925

**Published:** 2025-02-13

**Authors:** Lifang Guo, Imo Kalu Agwu, Umar Ishtiaq, Khalid A. Alnowibet

**Affiliations:** 1 School of Mathematics and Statistics, Chongqing Three Gorges University, Wanzhou, China; 2 Department of Mathematics, Micheal Okpara University of Agriculture, Umudike, Umuahia, Abia State, Nigeria; 3 Office of Research, Innovation and Commercialization, University of Management and Technology, Lahore, Pakistan; 4 Statistics and Operations Research Department, College of Science, King Saud University, Riyadh, Kingdom of Saudi Arabia; University of Education, PAKISTAN

## Abstract

In this paper, we introduce a novel class of nonlinear mappings known as *ϑ*-strictly asymptotically pseudocontractive-type multivalued mapping (*ϑ*-SAPM) in a Hilbert space domain. In addition, a new method was initiated, and it was shown that this method converges strongly to the solution set of an equilibrium problem (EP) and the set of common fixed points of two finite families of type-one (*ϑ*-SAPM) and *ϑ*-strictly pseudocontractive-type multivalued mapping (*ϑ*-SPM). Moreover, we showed that the classes of mappings considered are independent and also presented a numerical example to illustrate the implementablity of the suggested method. The results obtained improve, generalize and extend several conclusions reported in literature.

## 1 Introduction

In a fast developing area of nonlinear theory of differential equation, control theory, image recovery, game theory, etc,. many indispensable results have been established by the use of nonlinear functional analysis based on fixed point theory. In the recent past, fixed point theory has grown into a full fledged research area. Several notions associated with fixed point theory, which can be used to generate a particular elegant approach for the solution of nonlinear problems arising in mathematics, statistics, engineering, economics, approximation theory, theory of differential equations, theory of integral equations, etc., has been established in the contemporary literature (see, for example, [1] and the references therein).

After the initial impetus provided by Nadler [2] in 1969, unwavering attention has been given to the study of fixed point theorems for multivalued mappings. This stem from the significance of fixed point theory for this class of nonlinear mappings in different fields. Other interesting results that followed the remarkable conclusion obtained in [2] with respect to multivalued contraction mappings include, but not limited to the following: Markin [3] initiated the concept of employing Hausdorff metric to examine the fixed points of certain multivalued contraction and nonexpansive mappings, Hu et al [4] proved theorems concerning common fixed points of two multivalued nonexpansive mappings satisfying appropriate contractive inequalities, Bunyawat and Suntain [5] originated a method of establishing common element of solution for a countable family of multivalued quasi-nonexpansive mappings in a uniformly convex Banach space, Isogugu [6] initiated the concept of type-one *ϑ*-SPM which assures strong convergence without imposing any condition on the fixed point set, Agwu and Igbokwe [7] introduced a technique for obtaining common element of solution for minimization problems with fixed point constraint, etc.

However, we were being captivated by the following techniques studied in [9]: Let *H* be a real Hilbert space and ∅ ≠ *Q* ⊂ *H* be convex and closed. Given the point ℘_0_ ∈ *Q* and for each $c_{q,\xi^{o}}\in [0,1]$, let ${\left\{{ℑ}_{\xi^{o}}\right\}}_{\xi^{o}=1}^{\upsilon }:Q\to P\left(Q\right)$ (2≤υ∈N is a countable family of type-one $\vartheta_{\xi^{o}}$-SPM (defined below). The Mann-type technique developed by {℘_*q*_} is

$$\begin{array}{c} {℘}_{q+1}=c_{q,1}{℘}_{q}+\sum_{\xi^{o}=1}^{\upsilon }c_{q,\xi^{o}}\prod_{\tau =1}^{\xi^{o}-1}\left(1-c_{q,\tau }\right)\hbar_{q,\xi^{o}-1}+\prod_{\tau =1}^{\upsilon }\left(1-c_{q,\tau }\right)\hbar_{q,\upsilon },\;q\geq 1, \end{array}$$ (1)

where $\hbar_{q,\xi^{o}}\in {ℑ}_{\xi^{o}{℘}_{q}}$ for each *ξ*^*o*^. If f:Q×Q→R is a bifunction fulfilling (*B*1) − (*B*4) and ${\left\{{ℑ}_{\xi^{o}}\right\}}_{\xi^{o}=1}^{N}$ be as described above for each $\xi^{o}=1,2,\cdots ,N$, then the modified lshikawa technique developed from {℘_*q*_} is given by

$$\begin{array}{c} \left\{ \begin{array}{cc} \hbar_{q}=c_{q,1}{℘}_{n}+\sum_{\xi^{o}=1}^{\upsilon }c_{q,\xi^{o}}\prod_{j=1}^{\xi^{o}-1}\left(1-c_{q,\tau }\right)\hbar_{q,\xi^{o}-1}+\prod_{j=1}^{\upsilon }\left(1-c_{q,\tau }\right)\hbar_{q,\upsilon }; & \\ u_{q}\in Q:F\left(u_{q},\hbar \right)+\frac{1}{r_{q}}\left\langle \hbar -u_{q},u_{q}-\hbar_{q}\right\rangle \geq 0,\;\forall \hbar \in Q; & \\ {℘}_{q+1}=\frac{1}{2}\left(u_{q}+{℘}_{q}\right), \end{array}\right. \end{array}$$ (2)

where for some *a* > 0, {*r*_*q*_} ⊂ [*a*, ∞).

Our captivation is basically because of the introduction of the new schemes ((1) and (2)) that address the setbacks (sum conditions) which restricted the application of many results published in this direction.

Subsequently, an unwavering attention has be drawn to methods incorporating several auxiliary maps (see, for example, [8] for details) which is known to be more robust against certain numerical errors as compared to those that involve only one auxiliary mapping. In view of this, the following question becomes necessary:

**Question 1.1** *Can we obtain a method involving several auxiliary mapping which guarantees strong convergence for certain class of multivalued mappings?*

Moltivated and inspired by several works studied, and in particular the remarkable conclusions in [9], our focus in this paper are the following:

(*a*) To intiate the notion of *ϑ*-SAPM in a real Hilbert space domain;
(*b*) To address the request of Question 1.1 above.
(*c*) To establish strong convergence theorem involving equilibrium problems and mixed-type fixed point problems.

## 2 Relevant preliminaries

In what follows, the following concepts and known results will be required in order to prove our main results: Let *H* be a real Hilbert space *H* with the inner product 〈, ., 〉 and the norm ‖.‖ and ∅ ≠ *Q* ⊂ *H* be a convex and closed. Throughout the remaining sections in this paper, the following symbols shall be used: N will represent the set of natural numbers, R will represent the set of real numbers and ⇀ and → will represent weak and strong convergence of any sequence ${\left\{{℘}_{n}\right\}}_{n=1}^{\infty }$ in *H*, respectively.

Let ℑ, ð: *Q* → *Q* be two nonlinear mappings. We shall use *F*(ℑ), *F*(ð) and F={ℏ∈Q:ℏ∈ℑ∩ð} to denote the set of fixed points of ℑ and ð and the set common fixed point of ℑ and ð, respectively.

**Definition 2.1** *Recall that*

(*a*) ℑ *is known as an asymptotically strict pseudocontraction (ASPM, for short) if* $\exists {\left\{\nu_{q}\right\}}_{q=0}^{\infty }\subseteq \left[1,\infty \right):\underset{q\to \infty }{\text{lim}}\nu_{q}=1$ *and a ϑ* ∈ [0, 1) *that guarantees*
  $$\begin{array}{c} \|{ℑ}^{q}℘-{ℑ}^{q}{\hbar \|}^{2}\leq \nu_{q}^{2}{\left\|℘-\hbar \right\|}^{2}+\vartheta {\left\|\left(I-{ℑ}^{q}\right)℘-\left(I-{ℑ}^{q}\right)\hbar \right\|}^{2},\;\forall ℘,\hbar \in Q,\;q\geq 1. \end{array}$$ (3)
  *The class of mappings represented by* (3) *is a superclass of the class of asymptotically nonexpansive mappings (ANM, for short) (where* ℑ *is known as ANM if for all* ℘, ℏ ∈ *Q*, $\exists {\left\{\nu_{q}\right\}}_{q=1}^{\infty }\subseteq \left[1,\infty \right):\underset{q\to \infty }{\text{lim}}\nu_{q}=1$ *which assures the inequality* ‖ℑ^*q*^℘ − ℑ^*q*^ℏ‖ ≤ *ν*_*q*_‖℘ − ℏ‖, ∀*q* ≥ 1) *studied in* [10].
  **Remark 2.1** *It is worthy to mention that if F*(ℑ) ≠ ∅, *then* (3) *becomes an asymptotically demicontractive mapping (ADM, for short)*.
(*b*) ℑ *is known as k-strictly pseudocontractive if there exists a constant ϑ* ∈ [0, 1) *such that for all* ℘, ℏ ∈ *Q, we have*
  $$\begin{array}{c} {\left\|ℑ℘-ℑ\hbar \right\|}^{2}\leq {\left\|℘-\hbar \right\|}^{2}+\vartheta {\left\|\left(I-ℑ\right)℘-\left(I-ℑ\right)\hbar \right\|}^{2},\;q\geq 1. \end{array}$$ (4)
  *This class of k-strictly pseudocontractive has been extensively studied by several authors (see, for example*, [7, 8, 11, 12] *and the reference therein). It is shown in* [13] *that a strictly pseudocontractive map is L Lipschitzian (i.e*., ‖ℑ℘ − ℑℏ‖ ≤ *L*‖℘ − ℏ‖ *for all* ℘, ℏ ∈ *D*(ℑ)) *in* [14] *that the class of k-strictly asymptotically pseudocontractive maps and the class of strictly pseudocontractive maps are independent*.
(*c*) ℑ *is called uniformly L-Lipschitzian if there exists a constant L* > 0 *such that*
  $$\begin{array}{c} \|{ℑ}^{q}℘-{ℑ}^{q}\hbar \|\leq L\|℘-\hbar \|\;\forall ℘,\hbar \in K,\forall q\in N, \end{array}$$
  *and is said to be demiclosed at a point ν if whenever* ${\left\{{℘}_{n}\right\}}_{n=1}^{\infty }$ *is a sequence in D*(ℑ) *such that* ${\left\{{℘}_{n}\right\}}_{n=1}^{\infty }$ *converges weakly to* ℘^⋆^ ∈ *D*(ℑ) *and* ${\left\{ℑ{℘}_{n}\right\}}_{n=1}^{\infty }$ *converges strongly to ν, then* ℑ℘^⋆^ = *ν*.

Let ℧:Q×Q→R be a bifunction. An EP for ℧ is to search for an *ω* ∈ *Q* that assures the inequality

$$\begin{array}{c} ℧(\omega ,\hbar )\geq 0,\;\forall \hbar \in Q. \end{array}$$ (5)

A point *z* ∈ *Q* is referred to as an equilibrium point if it solves problem (5).

We shall use EP(℧) to indicate the solution set of problem (5); that is,

$$\begin{array}{c} EP(℧)=\{\omega \in Q:℧(\omega ,\hbar )\geq 0,\;\forall \hbar \in Q\} \end{array}$$ (6)

Considering the invaluable position of equilibrium problems in real life applications, several methods have been deployed to approximate the solution of problem (5); see [15] for more detail. In recent past, different authors have investigated joint problems involving equilibrium and fixed point problem of one mapping in the Hilbert space domain; see, for instance, [5, 9, 15–19] and the references contained in them.

Let *B* denote a strong positive bounded linear operator on a real Hilbert space domain *H*; that is, it is possible to get a constant $\bar{\alpha }>0$ which assures that inequality

$$\begin{array}{c} \left\langle B\hbar ,\hbar \right\rangle \geq \bar{\alpha }{\left\|\hbar \right\|}^{2},\;\forall y\in H. \end{array}$$

The problem here is to minimize a quadratic function over the set of fixed points of a nonexpansive mapping *τ* in a real Hilbert space domain:

$$\begin{array}{c} \underset{\mu \in F(ℑ)}{\text{min}}\frac{1}{2}\left\langle B\mu ,\mu \right\rangle -\left\langle \mu ,b\right\rangle , \end{array}$$

given that *b* ∈ *H*.

In view of the above, and motivated the results in [20], Marino and Xu [21] initiated the following method for approximating the fixed point of nonexpansive mapping via viscosity technique initiated by Moudafi [22]:

$$\begin{array}{c} \mu_{n+1}=\alpha_{q}\gamma f\left(\mu_{q}+\left(1-\alpha_{q}A\right)\right)ℑ\mu_{q},\;q\in N, \end{array}$$ (7)

where ℑ and *f* represent nonexpansive and contraction mappings, respectively. Using (7), they obtained a strong convergence to the unique solution of the variational inequality problem

$$\begin{array}{c} \langle \left(B-\gamma f\right)\mu^{⋆},\mu -\mu^{⋆}\rangle \geq 0,\;\forall \mu \in F\left(\tau \right), \end{array}$$ (8)

which represents the optimality condition for the minimization problem

$$\begin{array}{c} \underset{\mu \in F(ℑ)}{\text{min}}\frac{1}{2}\left\langle B\mu ,\mu \right\rangle -\varsigma \left(\mu \right), \end{array}$$

with ς denoting the potential function of *γf* (i.e., $\varsigma^{\prime }\left(\mu \right)=\gamma f\left(\mu \right),\forall \mu \in H)$.

Generally, approximating fixed points of single-valued mappings is simpler compared to its multivalued counterpart. However, several researchers have continued to investigate different methods of obtaining invariant point of multivalued mappings, reasons basically contained in their involvement in several real world applications including optimisation and variational inequalities problems (see [2, 3, 23–28]).

A subset *Q* of a normed space Δ is considered as being proximinal if it is possible to find a point *ϕ* ∈ *Q* which assures

$$\begin{array}{c} \rho (℘,Q)=\text{inf}\{\|℘-\hbar \|:\hbar \in Q\}=\rho (℘,\phi ). \end{array}$$ (9)

for each ℘ ∈ Δ. It has been established that a subset of a real uniformly convex Banach space admitting closedness and convexity properties and a subset of a real Banach space guaranteeing convexity and weakly compactness properties are both proximinal.

In what follows, *CB*(Δ), *C*(*Q*) *and* P(Q) shall represent the family of nonempty bounded closed subsets of Δ, the family of nonempty compact subsets of *Q* and the family of nonempty bounded proximinal subsets of *Q*, respectively. The Hausdorff metric induced by the metric *ρ* of Δ for all *A*, *B* ∈ *CB*(Δ) is given as

$$\begin{array}{c} \Theta \left(A,B\right)=\text{max}\;\{\underset{℘\in A}{\text{sup}}\;\rho \left(℘,B\right),\underset{\hbar \in B}{\text{sup}}\;\rho \left(\hbar ,A\right)\}, \end{array}$$ (10)

where *ρ*(℘, *B*) = inf{‖℘ − ℏ‖ : ℏ ∈ *B*} denotes the distance from the point ℘ to the subset *B*. A point ℘^⋆^ ∈ *Q* is said to be a fixed point of the multivalued mapping ℑ if ℘^⋆^ ∈ ℑ℘^⋆^. T Denote by *F*(ℑ) = {℘ ∈ *Q* : ℘ ∈ ℑ℘} the set of fixed points of ℑ

**Definition 2.2** *The* ℑ : *D*(ℑ) ⊆ Δ → 2^Δ^ *is known as*:

1. *uniformly L-Lipschitzian it it is possible to get an L* ≥ 0 *which assures*
  $$\begin{array}{c} \Theta \left({ℑ}^{n}℘,{ℑ}^{n}\hbar \right)\leq L\left\|℘-\hbar \right\|,\;\forall ℘,\hbar \in D\left(ℑ\right). \end{array}$$ (11)
  *If L* = *ν*_*q*_ *in* (11), *where* ${\left\{\nu_{q}\right\}}_{n=1}^{\infty }\subseteq \left[1,\infty \right):\underset{q\to \infty }{\text{lim}}\nu_{q}=1$, *then* ℑ *becomes ANM*.
2. *type-one* [6] *if* ∀℘, ℏ ∈ *D*(ℑ), *we get*
  $$\begin{array}{c} \left\|a-b\right\|\leq \Theta \left(ℑ℘,ℑ\hbar \right),\;\forall a\in P_{ℑ}℘,b\in P_{ℑ}\hbar , \end{array}$$ (12)
  *where* $P_{ℑ}℘=\left\{a\in ℑ℘:\|℘-a\|=\rho \left(℘,ℑ℘\right)\right\}$.
3. *ϑ-strictly asymptotically pseudocontraction* (*ϑ-SAPM*) *if it is possible to find a sequence* ${\left\{\nu_{q}\right\}}_{q=1}^{\infty }\subseteq \left[1,\infty \right):{\text{lim}}_{q\to \infty }\nu_{q}=1$ *and a k* ∈ [0, 1) *in which, for any pair* ℘, ℏ ∈ *D*(ℑ) *and an a* ∈ ℑ^*q*^℘, ∃*b* ∈ ℑ^*q*^ℏ *assuring* ‖*a* − *b*‖ ≤ *Θ*(ℑ^*n*^℘, ℑ^*q*^ℏ) *and*
  $$\begin{array}{c} \Theta {\left({ℑ}^{q}℘,{ℑ}^{q}\hbar \right)}^{2}\leq \nu_{q}\|℘-\hbar \|+\vartheta \|℘-a-\left(\hbar -b\right){\|}^{2},. \end{array}$$ (13)
  *If ϑ* = 1 *in* (13) *then* ℑ *becomes asymptotically pseudocontractive; whereas* ℑ *reduces to ANM if ϑ* = 0 *in* (13).

Very recently, Isogugu [29] introduced the following nonlinear map in the Hilbert space domain:

**Definition 2.3** *Let X be a normed space and* ℑ : *D*(ℑ) ⊆ *X* → 2^*X*^ *be a given map. Then* ℑ *is known as ϑ-strictly pseudocontractive-type in the sense of Browder and Petryshyn* [30] *if there exists ϑ* ∈ [0, 1) *such that given any* ℘, ℏ ∈ *D*(ℑ), *and a* ∈ ℑ℘, *we can find b* ∈ ℑℏ *satisfying* ‖*a* − *b*‖ ≤ *Θ*(ℑ℘, ℑℏ) *and*

$$\begin{array}{c} \Theta {\left(ℑ℘,ℑ\hbar \right)}^{2}\leq {\left\|℘-\hbar \|+\vartheta \|℘-a-\left(\hbar -b\right)\right\|}^{2}. \end{array}$$ (14)

*Note that* ℑ *in* (14) *becomes pseudocontractive-type if ϑ* = 1 *and nonexpansive-type if* *ϑ* = 0. *It is not hard to see from* (14) *that every nonexpansive-type multivalued mapping is ϑ-strictly pseudocontractive-type and every ϑ-strictly pseudocontractive type multivalued mapping is pseudocontractive-type. It is shown in* [29] *that the class of nonexpansive-type and ϑ-strictly pseudocontractive-type multivalued mappings are properly contained in the class of ϑ-strictly pseudocontractive-type and pseudocontractive-type multivalued mappings, respectively*.

**Definition 2.4** [6] *Let E be a Banach space and* ℑ : *D*(ℑ) ⊆ *E* → 2^*E*^ *be a multivalued mapping*. *I* − ℑ *is said to be weakly demiclosed at zero if for any sequence* ${\left\{{℘}_{n}\right\}}_{n=1}^{\infty }\subseteq D\left(ℑ\right)$ *such that* {℘_*n*_} *converges weakly to ν and a sequence* ${\left\{\hbar_{n}\right\}}_{n=1}^{\infty }$ *with* ℏ_*n*_ ∈ ℑ℘_*n*_ *for all* n∈N *such that* {℘_*n*_ − ℏ_*n*_} *strongly converges to zero. Then, ν* ∈ ℑ*ν*(*i.e*., 0 ∈ (*I* − ℑ)*ν*).

**Lemma 2.1** 1001 [21] *Consider a bounded linear mapping A on H which assures strongly positive self adjoint (with the coefficient* *ϰ* > 0 *and* 0 < *ϱ* ≤ ‖*A*‖^−1^), *then* ‖1 − *ϱA*‖ ≤ 1 − *ϱϰ*.

**Lemma 2.2** 1001 [12] *Let H be as described above. Then*

$$\begin{array}{c} {\left\|℘-\hbar \right\|}^{2}\leq {\left\|℘\right\|}^{2}+2\left\langle \hbar ,℘+\hbar \right\rangle ,\;\forall ℘,\hbar \in H. \end{array}$$

**Lemma 2.3** (*see* 1001 [20]) *Let* {*φ*_*n*_} ⊂ [0, ∞) *with φ*_*n*+1_ = (1 − *α*_*n*_)*φ*_*n*_ + *σ*_*n*_, *n* ≥ 0, *where* {*α*_*n*_} ⊂ (0, 1) *and* {*σ*_*n*_} *is a sequence in R such that* $\sum_{n=0}^{\infty }\alpha_{n}=\infty$ *and* ${\text{lim}\;\text{sup}}_{n\to \infty }\frac{\sigma_{n}}{\alpha_{n}}\leq 0$. *Then*, lim_*n*→∞_ *φ*_*n*_ = 0.

**Lemma 2.4** 1001 [31] *For each* ℘_1_, ℘_2_, ⋯, ℘_*m*_ *and α*_1_, *α*_2_, ⋯, *α*_*m*_ ∈ [0, 1] *with* $\sum_{i=1}^{m}\alpha_{i}=1$, *we have*

$$\begin{array}{c} \|\alpha_{1}{℘}_{1}+\alpha_{2}{℘}_{2}+\cdots +\alpha_{m}{℘}_{m}{\|}^{2}=\sum_{i=1}^{m}\alpha_{i}\|{℘}_{i}{\|}^{2}-\sum_{0\leq i<j\leq m}\alpha_{i}\alpha_{j}{\left\|{℘}_{i}-{℘}_{j}\right\|}^{2}. \end{array}$$ (15)

**Lemma 2.5** 1001 [32] *Let* {ℏ_*r*_}_*r*≥1_ *be a sequence of real numbers that does not decrease at infinity. In addition, consider the sequence of integers* ${\left\{\phi_{\left(r\right)}\right\}}_{r\geq r_{0}}$ *defined by*

$$\begin{array}{c} \phi_{\left(r\right)}=\text{max}\left\{k\in N:k\leq r,\hbar_{k}\leq \hbar_{k+1}\right\}. \end{array}$$

*Then*, ${\left\{\phi_{\left(r\right)}\right\}}_{r\geq r_{0}}$ *is a nondecreasing sequence verifying* $\underset{r\to \infty }{\text{lim}}\phi_{\left(r\right)}=0$ *and for all* *r* ≥ *r*_0_, *the following two inequalities hold*:

$$\begin{array}{c} \hbar_{\phi (r)}\leq \hbar_{\phi (r)+1}\;\text{and}\;\hbar_{r}\leq \hbar_{\phi (r)+1}. \end{array}$$

For solving the equilibrium problem, we take the following assumptions into consideration: the function ℧ : *Q* × *Q* → *R* satisfies the following conditions:

(*M*1) ℧(℘,℘)=0,∀℘∈Q;
(*M*2) ℧ is monotone, i.e, ℧(℘,ℏ)+℧(ℏ,℘)≤0,∀℘,ℏ∈Q;
(*M*3) ℧ is upper hemicontinuous, i.e., for each ℘, ℏ, *z* ∈ *Q*,
  $$\begin{array}{c} \underset{t\to 0^{+}}{\text{lim}\;\text{sup}}℧\left(tz+\left(1-t\right)℘,\hbar \right)\leq ℧\left(℘,\hbar \right); \end{array}$$
(*B*4) ℧(℘,.) is convex and lower semicontinuous for each ℘ ∈ *Q*.

**Lemma 2.6** 1001 [33] *Let H be a real Hilbert space H*, ∅ ≠ *Q* ⊂ *H be closed and convex and let* ℧ *be a bifunction of Q* × *Q assuring* (*M*1) − (*M*4). *For r* > 0, *and given* ℘∈H, *we can find* z∈Q that guarantees the inequality

$$\begin{array}{c} ℧\left(℘,\hbar \right)+\frac{1}{r}\left\langle \hbar -z,z-℘\right\rangle \geq 0,\;\forall \hbar \in Q. \end{array}$$ (16)

**Lemma 2.7** 1001 [15] *Assume that* ℧:Q×Q *assures* (*B*1) − (*B*4). *Define an a operator* ℑ_*r*_ : *H* → *Q* *as*

$$\begin{array}{c} {ℑ}_{r}℘=\left\{z\in Q:℧\left(z,y\right)+\frac{1}{r}\left\langle \hbar -z,z-℘\right\rangle \geq 0,\;\forall \left(\hbar ,℘\right)\in Q\times H\right\}, \end{array}$$

*where r* > 0. *Subsequently*,

(*i*) ℑ_*r*_ *is single-valued*;
(*ii*) *for any* ℘, ℏ ∈ *H*,
  $$\begin{array}{c} \|{ℑ}_{r}℘-{ℑ}_{r}{\hbar \|}^{2}\leq \left\langle {ℑ}_{r}℘-{ℑ}_{r}\hbar ,℘-\hbar \right\rangle ; \end{array}$$
(*iii*) $F\left({ℑ}_{r}\right)=EP\left(℧\right);$

**Proposition 2.1** 1001 [9] *Let* ${\left\{\alpha_{\xi^{o}}\right\}}_{\xi^{o}=1}^{\infty }\subseteq N$ *be a countable subset of* R, *where s is a fixed nonnegative integer and υ is any integer with s* + 1 ≤ *υ*. *Then, the following identity holds*:

$$\begin{array}{c} \alpha_{s}+\sum_{\xi^{o}=s+1}^{\upsilon }(\alpha_{\xi^{o}}\prod_{q=s}^{\xi^{o}-1}\left(1-\alpha_{q}\right))+\prod_{q=s}^{\upsilon }\left(1-\alpha_{q}\right)=1. \end{array}$$ (17)

**Proposition 2.2** 1001 [9] *Let t*, *u*, *v* ∈ *H be arbitrary. Let s be any fixed nonnegetive integer and* υ∈N *be such that s* + 1 ≤ *υ*. *Let* ${\left\{v_{i}\right\}}_{t=1}^{\upsilon -1}\subseteq H$ *and* ${\left\{\alpha_{t}\right\}}_{t=1}^{\upsilon }\subseteq \left[0,1\right]$. *Define*

$$\begin{array}{c} \hbar =\alpha_{s}t+\sum_{\xi^{o}=s+1}^{\upsilon }(\alpha_{\xi^{o}}\prod_{q=s}^{\xi^{o}-1}\left(1-\alpha_{q}\right)v_{\xi^{o}-1})+\prod_{q=s}^{\upsilon }\left(1-\alpha_{q}\right)v. \end{array}$$

*Then,*

$$\begin{array}{ccc} {\left\|\hbar -u\right\|}^{2} & = & \alpha_{s}{\left\|t-u\right\|}^{2}+\sum_{\xi^{o}=s+1}^{\upsilon }(\alpha_{\xi^{o}}\prod_{q=s}^{\xi^{o}-1}\left(1-\alpha_{q}\right){\left\|v_{\xi^{o}-1}-u\right\|}^{2})+\prod_{\tau =k}^{\upsilon }\left(1-\alpha_{q}\right){\left\|v-u\right\|}^{2}\\ & & -\alpha_{s}[\sum_{\xi^{o}=s+1}^{\upsilon }(\alpha_{\xi^{o}}\prod_{q=s}^{\xi^{o}-1}\left(1-\alpha_{q}\right){\left\|t-v_{\xi^{o}-1}\right\|}^{2})+\prod_{q=s}^{\xi^{o}-1}\left(1-\alpha_{q}\right){\left\|t-v\right\|}^{2}]\\ & & -\left(1-\alpha_{s}\right)[\sum_{\xi^{o}=s+1}^{\upsilon }(\alpha_{\xi^{o}}\prod_{q=s}^{\xi^{o}-1}\left(1-\alpha_{q}\right){\left\|v_{\xi^{o}-1}-\left(\alpha_{\xi^{o}+1}+w_{\xi^{o}+1}\right)\right\|}^{2})\\ & & +\alpha_{\upsilon }\prod_{\tau =k}^{\xi^{o}-1}\left(1-\alpha_{q}\right){\left\|v-v_{\upsilon -1}\right\|}^{2}], \end{array}$$

*where* $w_{s}=\sum_{\xi^{o}=s+1}^{\upsilon }(\alpha_{\xi^{o}}\prod_{q=s}^{\xi^{o}-1}\left(1-\alpha_{q}\right)v_{\xi^{o}-1})+\prod_{q=s}^{\xi^{o}-1}\left(1-\alpha_{q}\right)v,s=1,2,\cdots ,\upsilon$ *and w*_*q*_ = (1 − *c*_*q*_)*v*.

Recently, Rizwan et al. [34–38] worked on several types of fixed point algorithms, HR-Ciric-Reich-Rus contractions, generalized enriched contractions, and MR-Kannan-type interpolative contractions. They provide very important applications of fixed point theory including activation functions through fixed-circle problems.

## 3 Main results

**Definition 3.1** *Let X be a normed space and* ℑ : *D*(ℑ) ⊆ *X* → 2^*X*^ *be a given map. Then*, ℑ *is k-ASPM in the thought of Isogugu et al*. [9] *if there exists μ* ∈ [0, 1) *such that given any* ℘, ℏ ∈ *D*(ℑ) *and u*_*q*_ ∈ ℑ^*q*^℘, *we can find* ${\left\{\nu_{q}\right\}}_{q=1}^{\infty }\subseteq \left[1,\infty \right)$ *with* $\sum_{q=1}^{\infty }\left(\nu_{q}-1\right)<\infty$ *and v*_*q*_ ∈ ℑ^*q*^ℏ *satisfying* $\|u_{q}-v_{q}\left\|\right\|\leq D\left({ℑ}^{q}℘,{ℑ}^{q}\hbar \right)$ *for which*

$$\begin{array}{c} D^{2}\left({ℑ}^{q}℘,{ℑ}^{q}\hbar \right)\leq \nu_{q}^{2}{\left\|℘-\hbar \right\|}^{2}+k{\left\|℘-u_{q}-\left(\hbar -v_{q}\right)\right\|}^{2}. \end{array}$$ (18)

**Remark 3.1** *From Definition 3.1, it is not difficult to see that every multivalued nonexpansive-type mapping is strictly asymptotically pseudocontrctive-type mapping. The examples below show that the class multivalued nonexpansive-type mapping is properly included into the class of multivalued strictly asymptotically pseudocontrctive-type mapping and the class of multivalued strictly asymptotically pseudocontractive-type mapping is properly included into the class of asymptotically pseudocontrctive-type mapping*.

**Example 3.1** (*see* [39]) *Give* X=R *the usual metric and let the map* $ℑ:\left[0,\infty \right)\to 2^{R}$ *be given as*

$$\begin{array}{c} ℑ℘=[-\frac{℘}{\sqrt{5}},-\frac{℘}{\sqrt{3}}]. \end{array}$$

*Then, for n odd* (*q* ≥ 2), *we obtain*

$$\begin{array}{c} {ℑ}^{q}℘=[-\frac{℘}{\sqrt{3^{2q-1}}},-\frac{℘}{\sqrt{5^{2q-1}}}]. \end{array}$$

*Now*,

$$\begin{array}{ccc} D^{2}\left({ℑ}^{q}℘,{ℑ}^{q}\hbar \right) & = & \text{max}\{|\frac{1}{\sqrt{3^{2q-1}}}\left(℘-\hbar \right){|}^{2},|\frac{1}{\sqrt{5^{2q-1}}}\left(℘-\hbar \right){|}^{2}\}\\ & = & \frac{1}{3^{2q-1}}{\left||℘-\hbar \right|}^{2}\\ & < & \frac{1}{3^{2q-3}}{\left||℘-\hbar \right|}^{2}\\ & = & (\frac{9}{3^{2q}}+\frac{17}{3^{2q-1}}){\left||℘-\hbar \right|}^{2}+\frac{1}{3^{2q-1}}{\left|℘-\hbar \right|}^{2}\\ & < & (\frac{9}{3^{2}}+\frac{17}{3^{2q-1}}){\left||℘-\hbar \right|}^{2}+\frac{1}{3^{2}}{\left|℘-\hbar \right|}^{2}. \end{array}$$ (19)

*Also, for each* $u_{q}\in {⅁}^{q}℘,u_{q}=-\delta^{q}℘,\frac{1}{\sqrt{5^{2q-1}}}\leq \delta^{n}\leq \frac{1}{\sqrt{3^{2q-1}}}$. *Choose v*_*q*_ = −*δ*^*q*^ℏ. *Then*

$$\begin{array}{c} |u_{q}-v_{q}|=|-\delta ℘-\left(-\delta \hbar \right)|=|\delta \left(℘-\hbar \right)|\leq \frac{1}{\sqrt{3^{2q-1}}}\left|℘-\hbar \right|=D\left({⅁}^{n}℘,{⅁}^{q}\hbar \right) \end{array}$$

*and*

$$\begin{array}{c} |℘-u_{q}-\left(\hbar -v_{q}\right){|}^{2}=|℘-\delta ℘-\left(\hbar -\left(-\delta \hbar \right)\right){|}^{2}=|\left(1+\delta^{n}\right)℘-\left(1+\delta^{q}\right){\hbar |}^{2}={\left(1+\delta^{q}\right)}^{2}{\left|℘-\hbar \right|}^{2}.\; \end{array}$$ (20)

*From* (19) *and* (20), *we obtain*

$$\begin{array}{ccc} D^{2}\left({⅁}^{q}℘,{⅁}^{q}\hbar \right) & \leq & (\frac{9}{3^{2}}+\frac{17}{3^{2q-1}}){\left|℘-\hbar \right|}^{2}+\frac{1}{3^{2}}{\left|℘-\hbar \right|}^{2}\\ & = & (\frac{9}{3^{2}}+\frac{17}{3^{2q-1}}){\left||℘-\hbar \right|}^{2}+\frac{{\left(1+\delta^{q}\right)}^{2}}{3^{2}{\left(1+\delta^{q}\right)}^{2}}{\left|℘-\hbar \right|}^{2}\\ & = & (\frac{9}{3^{2}}+\frac{17}{3^{2q-1}}){\left||℘-\hbar \right|}^{2}+\frac{1}{3^{2q-1}{\left(1+\delta \right)}^{2}}{\left|℘-u_{q}-\left(\hbar -v_{q}\right)\right|}^{2}\\ & \leq & (\frac{9}{3^{2}}+\frac{17}{3^{2q-1}}){\left||℘-\hbar \right|}^{2}+\frac{1}{3^{2}{\left(1+\frac{1}{\sqrt{5^{2q-1}}}\right)}^{2}}{\left|℘-u_{q}-\left(\hbar -v_{q}\right)\right|}^{2}\\ & < & (\frac{9}{3^{2}}+\frac{17}{3^{2q-1}}){\left||℘-\hbar \right|}^{2}+\frac{1}{3^{2}{\left(1+\frac{1}{5}\right)}^{2}}{\left|℘-u_{q}-\left(\hbar -v_{q}\right)\right|}^{2}\\ & = & (1+\frac{17}{3^{2q-1}}){\left||℘-\hbar \right|}^{2}+\frac{25}{324}{\left|℘-u_{q}-\left(\hbar -v_{q}\right)\right|}^{2}. \end{array}$$

The following example shows that the class of *θ*-strictly asymptotically pseudocontractivetype multivalued mapping is more general than the class of asymptotically nonexpansive-type mappings.

**Example 3.2** *Let* H=R *be endowed with the usual metric and define the mapping* $ℑ:\left[-1.5,1\right]\to 2^{R}$ *by*

$$\begin{array}{c} ℑ℘=\{\begin{array}{c} \left[-\frac{2}{3}℘,-\frac{3}{2}℘],\;℘\in [-\frac{3}{2},0\right]\\ {}[-\frac{3}{2}℘,-\frac{2}{3}℘],\;℘\in \left[0,1\right] \end{array} \end{array}$$

*Then, for n odd q* ≥ 2, *we get*

$$\begin{array}{c} {ℑ}^{q}℘=\{\begin{array}{c} \left[-\frac{2}{3}℘,-\frac{3}{2}℘],\;℘\in [-\frac{3}{2},0\right]\\ {}[-\frac{3}{2}℘,-\frac{2}{3}℘],\;℘\in \left[0,1\right] \end{array} \end{array}$$

*Then*, $P_{{ℑ}^{q}}℘=\{-\frac{3}{2}℘\}$ *for all* ℘ ∈ [−1.5, 1] *and hence it is not ANM. Indeed*,

$$\begin{array}{c} D\left({ℑ}^{q}℘,{ℑ}^{q}\hbar \right)=\|P_{{ℑ}^{q}}℘-P_{{ℑ}^{q}}\hbar \|=|-\frac{3}{2}℘-(-\frac{3}{2}\hbar )|=\frac{3}{2}\left|℘-\hbar |>|℘-\hbar \right| \end{array}$$

*Observe that for each* $u_{n}\in {ℑ}^{q}℘,u_{q}=-\delta^{q}℘,\frac{2}{3}\leq \delta^{q}\leq \frac{3}{2}$ . *Choose v*_*q*_ = −*δ*^*q*^ℏ *so that*

$$\begin{array}{c} |u_{q}-v_{q}|=|-\delta ℘-\left(-\delta \hbar \right)|=|\delta \left(℘-\hbar \right)|\leq \frac{3}{2}\left|℘-\hbar \right|=D\left({⅁}^{q}℘,{⅁}^{q}\hbar \right) \end{array}$$

*and*

$$\begin{array}{c} |℘-u_{q}-\left(\hbar -v_{q}\right){|}^{2}=|℘-\delta^{q}℘-\left(\hbar -\left(-\delta^{q}\hbar \right)\right){|}^{2}=|\left(1+\delta^{q}\right)℘-\left(1+\delta^{q}\right){\hbar |}^{2}={\left(1+\delta^{q}\right)}^{2}{\left|℘-\hbar \right|}^{2}.\; \end{array}$$ (21)

*Now,*

$$\begin{array}{ccc} D^{2}\left({ℑ}^{q}℘,{ℑ}^{q}\hbar \right) & = & \text{max}\{|\frac{2}{3}\left(℘-\hbar \right){|}^{2},|\frac{3}{2}\left(℘-\hbar \right){|}^{2}\}\\ & = & \frac{9}{4}{\left|℘-\hbar \right|}^{2}\\ & = & {\left|℘-\hbar \right|}^{2}+\frac{5}{4}{\left|℘-\hbar \right|}^{2}\\ & = & {\left|℘-\hbar \right|}^{2}+\frac{5{\left(1+\delta^{q}\right)}^{2}}{4{\left(1+\delta^{q}\right)}^{2}}{\left|℘-\hbar \right|}^{2}\\ & = & {\left|℘-\hbar \right|}^{2}+\frac{5}{4{\left(1+\delta^{q}\right)}^{2}}{\left|℘-u_{q}-\left(\hbar -v_{q}\right)\right|}^{2}\\ & \leq & {\left|℘-\hbar \right|}^{2}+\frac{5}{4{\left(1+\frac{2}{3}\right)}^{2}}{\left|℘-u_{q}-\left(\hbar -v_{q}\right)\right|}^{2}\\ & = & {\left|℘-\hbar \right|}^{2}+\frac{9}{20}{\left|℘-u_{q}-\left(\hbar -v_{q}\right)\right|}^{2} \end{array}$$

Therefore, ℑ is *k*-SAPM with *k*_*n*_ = 1 and $k=\frac{9}{20}$. Note that ℑ, not being ANM, demonstrates the conclusion that the class of ANM mappings is properly included into the class of *k*-SAPM.

Now, we show with the following examples that the class of multivalued asymptotically strictly pseudocontractive-type mappings and the class of multivalued strictly pseudocontractive-type mappings are independent.

**Example 3.3** *Let* H=R *be endowed with the usual metric and define* $ℑ:\left[0,\infty \right)\to 2^{H}$ *by*

$$\begin{array}{c} ℑ℘=[-\frac{5}{2}℘,2℘]. \end{array}$$

*It is shown in* [29] *that* ℑ *is a strictly pseudocontractive-type mapping*.

*For q even* (*q* > 1), *we have*

$$\begin{array}{c} {ℑ}^{q}℘=[{\left(\frac{5}{2}\right)}^{q}℘,2^{q}℘]. \end{array}$$

*Observe that for each* $u_{q}\in {ℑ}^{q}℘,u_{q}=\delta^{q}℘,2^{q}\leq \delta^{q}\leq {\left(\frac{5}{2}\right)}^{q}$. *Choose v*_*q*_ = *δ*^*q*^ℏ *so that*

$$\begin{array}{c} \|u_{q}-v_{q}\left\|=\right|\delta^{q}℘-\delta^{q}\hbar |=|\delta^{q}\left(℘-\hbar \right)|\leq {\left(\frac{5}{2}\right)}^{q}\left|℘-\hbar \right|=D\left({ℑ}^{q},{ℑ}^{q}\right) \end{array}$$

*and*

$$\begin{array}{c} \|℘-u_{q}-\left(\hbar -v_{q}\right){\|}^{2}=|℘-\delta^{q}℘-\left(\hbar -\delta^{q}\hbar \right){|}^{2}=|\left(1-\delta^{q}\right)\left(℘-\hbar \right){|}^{2}={\left(1-\delta^{q}\right)}^{2}{\left|℘-\hbar \right|}^{2}. \end{array}$$ (22)

*Now,*

$$\begin{array}{ccc} D^{2}\left({ℑ}^{q},{ℑ}^{q}\right) & = & \text{max}\{{\left(\frac{5}{2}\right)}^{q}{\left|℘-\hbar \right|}^{2},2^{q}{\left|℘-\hbar \right|}^{2}\}\\ & = & {\left(\frac{5}{2}\right)}^{q}{\left|℘-\hbar \right|}^{2}>\frac{5}{2}{\left|℘-\hbar \right|}^{2}\\ & = & {\left|℘-\hbar \right|}^{2}+\frac{3}{2}{\left|℘-\hbar \right|}^{2}\\ & = & {\left|℘-\hbar \right|}^{2}+\frac{3{\left(1-\delta^{q}\right)}^{2}}{2{\left(1-\delta^{q}\right)}^{2}}{\left|℘-\hbar \right|}^{2}\\ & = & {\left\|℘-\hbar \right\|}^{2}+\frac{3}{2{\left(1-\delta^{q}\right)}^{2}}{\left\|℘-u_{q}-\left(\hbar -v_{q}\right)\right\|}^{2}\\ & \geq & {\left\|℘-\hbar \right\|}^{2}+\frac{3}{2{\left(1-2^{q}\right)}^{2}}{\left\|℘-u_{q}-\left(\hbar -v_{q}\right)\right\|}^{2}\\ & > & {\left\|℘-\hbar \right\|}^{2}+\frac{3}{2^{2q+1}}{\left\|℘-u_{q}-\left(\hbar -v_{q}\right)\right\|}^{2}\\ & > & {\left\|℘-\hbar \right\|}^{2},\;\forall n\geq 2\\ & = & \nu_{q}{\left|℘-\hbar \right|}^{2}+k{\left\|℘-u_{q}-\left(\hbar -v_{q}\right)\right\|}^{2}, \end{array}$$

*where k* = 0 *and ν*_*q*_ = 1. *Hence*, ℑ *is not asymptotically k-strictly pseudocontractive-type.mapping*.

**Example 3.4** *Let* $H=\varsigma_{2}=\{\bar{℘}={\left\{{℘}_{i}\right\}}_{i=1}^{\infty }:{℘}_{i}\in K,\sum_{i=1}^{\infty }|{℘}_{i}{|}^{2}<\infty \}$ *and let* $\bar{B}\{\bar{℘}\in \varsigma_{2}:\|℘\|\leq 1\}$ . *Define* $ℑ:\bar{B}\to 2^{H}$ *by*

$$\begin{array}{c} ℑ\bar{℘}=\left[\left(0,0,0,0,\cdots \right),\left(0,{℘}_{1}^{2},a_{2}{℘}_{2},a_{3}{℘}_{2},\cdots \right)\right], \end{array}$$

*where* ${\left\{a_{i}\right\}}_{i=1}^{\infty }$ *is a real sequence satisfying a*_2_, *a*_3_ > 0, 0 < *a*_*t*_ < 1, *t* ≠ 2, 3 *and* $\prod_{t=2}^{\infty }a_{t}=\frac{1}{2}$. *Then*,

$$\begin{array}{ccc} D^{2}\left({ℑ}^{q}℘,{ℑ}^{q}\hbar \right) & \leq & 2(\prod_{t=2}^{q}a_{t}){\left\|\bar{℘}-\bar{\hbar }\right\|}^{2}\\ & \leq & 2(\prod_{t=2}^{q}a_{t})\|\bar{℘}-\bar{\hbar }{\|}^{2}+k{\left\|\bar{℘}-u_{q}-\left(\bar{\hbar }-v_{q}\right)\right\|}^{2}, \end{array}$$

*for all k* ∈ (0, 1), *n* ≥ 1 *and* $\bar{℘},\bar{\hbar }\in H$, *where* $u_{q}\in {⅁}^{q}℘,v_{q}\in {⅁}^{n}\hbar$. *Since* ${\text{lim}}_{n\to \infty }2(\prod_{t=2}^{\infty }a_{t})=1$, *it follows that* ℑ *is asymptotically pseudocontractive-type*.

*Now, choose* $\bar{℘}=(\frac{1}{3},\frac{1}{3},\frac{1}{3},0,0,\cdots ),\bar{\hbar }=\left(0,0,0,0,\cdots \right),a_{2}=3$ *and a*_3_ = 4, *then we get*

$$\begin{array}{ccc} D^{2}\left({ℑ}^{q}℘,{ℑ}^{q}\hbar \right) & = & \|(0,\frac{1}{9},1,\frac{4}{3},\cdots ){\|}^{2}\\ & = & \left\langle (0,\frac{1}{9},1,\frac{4}{3},\cdots ),(0,\frac{1}{9},1,\frac{4}{3},\cdots )\right\rangle \\ & = & \frac{225}{81}>\frac{1}{9}+\frac{130}{81}k\\ & = & \|\bar{℘}-\bar{\hbar }{\|}^{2}+k{\left\|\bar{℘}-u-\left(\bar{\hbar }-v\right)\right\|}^{2}, \end{array}$$

*where* $u\in ℑ\bar{℘}$ *and* $v\in ℑ\bar{\hbar }$. *Hence*, ℑ *is not strictly pseudocontractive-type*.

Now, we shall prove the strong convergence of the new method to the solution set of an equilibrium problem (EP) and the set of common fixed points of two finite families of type-one (*θ*-SAPM) and *θ*-strictly pseudocontractive-type multivalued mapping (*θ*SPM).

**Theorem 3.1** *Let H*, *Q* *and* ℧ *be as described above. Suppose* ${\left\{{ð}_{\xi^{o}}\right\}}_{\xi^{o}=1}^{N};:Q\to P\left(Q\right)$ *and* ${\left\{{ℑ}_{\xi^{o}}\right\}}_{\xi^{o}=1}^{N}:Q\to P\left(Q\right)$, *υ* ≥ 2 *are finite families of type-one and* $L_{\xi^{o}}$-*uniformly Lipschitizian strictly asymptotically pseudocontractive-type and type-one strictly pseudocontractive-type multivalued mappings, respectively, with contractive coeficient* $k_{\xi^{o}}^{\left(1\right)},k_{\xi^{o}}^{\left(2\right)}\in \left[0.1\right)$ *for each* *ξ*^*o*^. *Suppose* $F=(\cap_{\varsigma =1}^{N}\left(F\left({ℑ}_{\varsigma }\right)\right)\cap \left(\cap_{\xi^{o}=1}^{N}F\left({ð}_{\xi^{o}}\right)\right)\cap EP\left(\Theta \right)\neq \emptyset$ *and for each* *ς*, $\left(I-{ℑ}_{\varsigma }\right)$ *and* $\left(I-{ð}_{\xi^{o}}\right)$ *are weakly demiclosed at zero. let* $f_{\xi_{o}}$ *be a ρ-contraction self map of Q with ρ* ∈ (0, 1) *and A be a strong positive self adjoint bounded linear operator on H with coeficient* $\bar{\alpha }$ *such that* $0<\rho \gamma +2\epsilon <\bar{\alpha }$. *Let* ${\left\{{℘}_{q}\right\}}_{n=0}^{\infty }$ *be a sequence developed from an arbitrary* ℘_0_ ∈ *Q by*

$$\begin{array}{c} \left\{ \begin{array}{cc} u_{q}∍℧\left(u_{q},\hbar \right)+\frac{1}{r_{q}}\left\langle \hbar -u_{q},u_{q}-{℘}_{q}\right\rangle \geq 0,\forall \hbar \in Q; & \\ \omega_{q}=\zeta_{q,1}u_{q}+\sum_{\xi^{o}=2}^{\upsilon }\zeta_{q,\xi^{o}}\prod_{\tau =1}^{\xi^{o}-1}\left(1-\zeta_{q,\tau }\right)\vartheta_{q,\xi^{o}-1}+\prod_{\tau =1}^{\upsilon }\left(1-\zeta_{q,\tau }\right)\vartheta_{q,N}; & \\ s_{q}={\beta^{o}}_{q,1}\omega_{q}+\sum_{\xi^{o}=2}^{\upsilon }{\beta^{o}}_{q,\xi^{o}}\prod_{\tau =1}^{\xi^{o}-1}\left(1-{\beta^{o}}_{q,\tau }\right)\pi_{q,\xi^{o}-1}+\prod_{\tau =1}^{\upsilon }\left(1-{\beta^{o}}_{q,\tau }\right)\pi_{q,\upsilon }; & \\ {℘}_{q+1}=P_{Q}\left(\alpha_{q}\gamma f\left({℘}_{q}\right)+\delta_{q}s_{q}+\left(\left(1-\delta_{q}\right)I-\alpha_{q}A\right)s_{q}\right), \end{array}\right. \end{array}$$ (23)

*where* $\vartheta_{q,\xi^{o}}\in {ℑ}_{\xi^{o}}u_{q}$ *and* $\pi_{q,\xi^{o}}\in {ð}_{\xi^{o}}^{n}\omega_{q}$ *for each* *ξ*^*o*^, {*α*_*q*_}, {*δ*_*q*_} ∈ [0, 1], ${\left\{{\left\{{\beta^{o}}_{q,\varsigma }\right\}}_{q=1}^{\infty }\right\}}_{\xi^{o}=1}^{\upsilon },{\left\{{\left\{\zeta_{q,\xi^{o}}\right\}}_{q=1}^{\infty }\right\}}_{\xi^{o}=1}^{\upsilon }\subseteq \left[0,1\right]$. *Suppose the requirements below are fulfilled*:

(*i*) ${\beta^{o}}_{q,1}\geq \zeta_{q,1}>max{\left\{k_{\xi^{o}}\right\}}_{\xi^{o}=1}^{\upsilon }:{\beta^{o}}_{q,\xi^{o}}\leq \zeta_{q,\xi^{o}}<\gamma \leq \beta^{o}<1,$ *for each i*;
(*ii*) $\underset{q\to \infty }{\text{lim}\;\text{inf}}\;{\beta^{o}}_{q,\xi^{o}}\prod_{j=1}^{\xi^{o}-1}\;\left(1-{\beta^{o}}_{q,\tau }\right)\left({\beta^{o}}_{q,1}-k_{\xi^{o}-1}\right)>0$ *and* $\underset{q\to \infty }{\text{lim}\;\text{inf}}\;\prod_{j=1}^{\xi^{o}-1}\left(1-{\beta^{o}}_{q,\tau }\right)\left({\beta^{o}}_{q,1}-k_{\upsilon }\right)>0;$
(*iii*) $\underset{q\to \infty }{\text{lim}\;\text{inf}}\;\zeta_{q,\xi^{o}}\prod_{j=1}^{\varsigma -1}\left(1-\zeta_{q,\tau }\right)\left(\zeta_{q,1}-k_{\xi^{o}-1}\right)>0$ *and* $\underset{q\to \infty }{\text{lim}\;\text{inf}}\;\zeta_{q,\xi^{o}}\prod_{\tau =1}^{\xi^{o}-1}\left(1-{\beta^{o}}_{q,j}\right)\left(\zeta_{q,1}-k_{N}\right)>0;$
(*iv*) $\underset{q\to \infty }{\text{lim}}\alpha_{q}=0,\sum_{n=1}^{\infty }\alpha_{q}=\infty$ *and* $\underset{q\to \infty }{\text{lim}}\frac{\left(1-{\beta^{o}}_{q,\tau }\right)\left(\nu_{q}^{2}-1\right)}{\alpha_{q}}=0;$
(*v*) {*r*_*q*_} ⊂ [*a*, ∞) *for some a* > 0.

*Then, the sequence* ${\left\{{℘}_{q}\right\}}_{n=0}^{\infty }$ *given by* (23) *admits strong convergence to* ϱ∈F , *which provides a solution to* 〈(A-γg)q,x-q〉≥0,∀x∈F.

To start with, we establish the fact that the operator $P_{F}\left[\delta I+\left(\alpha \gamma f+\left(\left(1-\delta \right)I-\alpha A\right)\right)\right]$ is a self contraction map of *Q*. Given $\alpha ,\delta \in (0,min\left\{1,\frac{1}{\bar{\alpha }}\right\})$ and for all ℘, ℏ ∈ *H*, it follows from Lemma 2.5 with $\lambda =P_{F}\left[\delta I+\left(\alpha \gamma f+\left(\left(1-\delta \right)I-\alpha A\right)\right)\right]℘$ and $\kappa =P_{F}\left[\delta I+\left(\alpha \gamma f+\left(\left(1-\delta \right)I-\alpha A\right)\right)\right]\hbar$ that

$$\begin{array}{ccc} \|P_{F}\lambda -P_{F}\kappa \| & \leq & \|P_{F}[\delta I+(\alpha \gamma f\\ & + & \left(\left(1-\delta \right)I-\alpha A\right))]℘-P_{F}\left[\delta I+\left(\alpha \gamma f+\left(\left(1-\delta \right)I-\alpha A\right)\right)\right]\hbar \|\\ & \leq & \alpha \gamma \|f(℘)-f(\hbar )\|+\delta \|℘-\hbar \|+\|((1-\delta )I-\alpha A)(℘-\hbar )\|\\ & \leq & \alpha \gamma \rho \|℘-\hbar \|+\delta \|℘-\hbar \|+(\left(1-\delta \right)I-\alpha \bar{\alpha })\|℘-\hbar \|\\ & = & (1-\left(\bar{\alpha }-\gamma \rho \right)\alpha ))\|℘-\hbar \|. \end{array}$$ (24)

Therefore, we can find a unique point ℘^⋆^ ∈ *Q* for which ${℘}^{⋆}=P_{F}\left[\delta I+\left(\alpha \gamma f+\left(\left(1-\delta \right)I-\alpha A\right)\right)\right]{℘}^{⋆},$ which we can write as

$$\begin{array}{c} \langle \left(I-A+\gamma f\right){℘}^{⋆}-{℘}^{⋆},{℘}^{⋆}-\nu \rangle \geq 0,\forall \nu \in F \end{array}$$

Since *α*_*q*_ → 0 as *q* → ∞, we can take $\alpha_{q}\in (0,\frac{1}{\left\|A\right\|})$ ∀*q* ≥ 0. Using condition (iv), it is possible to get a constant *ϵ* with 0 < *ϵ* < 1 − *δ* and $\sum_{\xi^{o}=2}^{\upsilon }{\beta^{o}}_{q,\xi^{o}}\prod_{\tau =1}^{\xi^{o}-1}\left(1-{\beta^{o}}_{q,\tau }\right)\left(\nu_{q}^{2}-1\right)<\epsilon \alpha_{q}$ for each *ς*. Also, by Lemma 2.1, we get $\|\left(1-\delta_{q}\right)I-\alpha_{q}A\|\leq \left(1-\delta_{q}\right)I-\alpha_{q}\bar{\alpha }$.

Let ${℘}^{⋆}\in F$ and $\nu_{q}=\text{max}\left\{\nu_{q,\varsigma }\right\},\varsigma =1,2,\cdots ,m$. Since $u_{q}=T_{r_{q}}{℘}_{q}$ and ${℘}^{⋆}=T_{r_{q}}{℘}^{⋆},$ we obtain (using Lemma 2.7) that

$$\begin{array}{c} \|u_{q}-{℘}^{⋆}\left\|\leq \right\|T_{r_{q}}{℘}_{q}-T_{r_{q}}{℘}^{⋆}\left\|\leq \right\|{℘}_{q}-{℘}^{⋆}\|. \end{array}$$ (25)

Further, we prove that ${\left\{{℘}_{q}\right\}}_{q=0}^{\infty }$ is bounded. Since ${ð}_{\varsigma }:Q\to P\left(Q\right)$ is *k*-SAPM and ${ℑ}_{\varsigma }:K\to P\left(K\right)$ *k*-SPM, *F*(ℑ) ≠ ∅ and F(ð)≠∅. Consequently, we can find a sequence ${\left\{{\left\{\nu_{q,\varsigma }\right\}}_{\varsigma =1}^{m}\right\}}_{q=1}^{\infty }\subseteq \left[1,\infty \right):\nu_{q,\varsigma }\to 1,\;for\;each\;\varsigma ,\;as\;q\to \infty$ and real positive constants $\sigma_{\varsigma }^{\left(1\right)},\sigma_{\varsigma }^{\left(2\right)}\in \left[0,1\right)$ such that for any $(℘\times {℘}^{⋆})\in Q\times F,$ we obtain

$$\begin{array}{c} \|\pi_{q,\varsigma }-{℘}^{⋆}{\|}^{2}\leq \nu_{q,\varsigma }\|{℘}_{q}-{℘}^{⋆}{\|}^{2}+\sigma_{i}^{\left(1\right)}{\left\|\omega_{n}-\vartheta_{n,i}\right\|}^{2},\;\pi_{q,\varsigma }\in {ð}_{\varsigma }^{q}\omega_{q} \end{array}$$ (26)

and

$$\begin{array}{c} \|\vartheta_{q,\varsigma }-{℘}^{⋆}{\|}^{2}\leq \|{℘}_{q}-{℘}^{⋆}{\|}^{2}+\sigma_{\varsigma }^{\left(2\right)}{\left\|u_{q}-\pi_{q,\varsigma }\right\|}^{2},\;\vartheta_{q,\varsigma }\in {ℑ}_{\varsigma }u_{q}. \end{array}$$ (27)

By (23) and Proposition 2.1 with *s*_*q*_ = *η*, *ω*_*q*_ = *t*, ℘^⋆^ = *u*, *s* = 1 and $\pi_{s,N}\in {ð}_{\varsigma }^{q}\omega_{q},$ we have

$$\begin{array}{ccc} \|s_{q}-{℘}^{⋆}{\|}^{2} & \leq & {\beta^{o}}_{q,1}{\left\|\omega_{q}-{℘}^{⋆}\right\|}^{2} \end{array}$$ (28)

$$\begin{array}{ccc} & + & \sum_{\xi^{o}=2}^{\upsilon }{\beta^{o}}_{q,\xi^{o}}\prod_{\tau =1}^{\xi^{o}-1}\left(1-{\beta^{o}}_{q,\tau }\right)\|\pi_{q,\xi^{o}-1}-{℘}^{⋆}{\|}^{2}+\prod_{\tau =1}^{\upsilon }\left(1-{\beta^{o}}_{q,\tau }\right){\left\|\pi_{q,\upsilon }-{℘}^{⋆}\right\|}^{2} \end{array}$$

$$\begin{array}{ccc} & - & {\beta^{o}}_{q,1}[\sum_{\xi^{o}=2}^{\upsilon }{\beta^{o}}_{q,\xi^{o}}\prod_{\tau =1}^{\xi^{o}-1}\left(1-{\beta^{o}}_{q,\tau }\right){\left\|\omega_{q}-\pi_{q,\xi^{o}-1}\right\|}^{2} \end{array}$$ (29)

$$\begin{array}{ccc} & + & \prod_{\tau =1}^{\upsilon }\left(1-{\beta^{o}}_{q,\tau }\right){\left\|\omega_{q}-\pi_{q,\upsilon }\right\|}^{2}] \end{array}$$ (30)

Since ${ð}_{\xi^{o}}$ is type-one *k*-SAPM, we have, using (30), that

$$\begin{array}{ccc} \|s_{q}-{℘}^{⋆}{\|}^{2} & \leq & {\beta^{o}}_{q,1}{\left\|\omega_{q}-{℘}^{⋆}\right\|}^{2}\\ & + & \sum_{\xi^{o}=2}^{\upsilon }{\beta^{o}}_{q,\xi^{o}}\prod_{\tau =1}^{\xi^{o}-1}\left(1-{\beta^{o}}_{q,\tau }\right)[{\left(\nu_{q,\xi^{o}}^{\left(1\right)}\right)}^{2}\|\omega_{q}-{℘}^{⋆}{\|}^{2}+\sigma_{\xi^{o}-1}^{\left(1\right)}\|\omega_{q}-\pi_{q,\xi^{o}-1}{\|}^{2}]\\ & + & \prod_{j=1}^{\upsilon }\left(1-{\beta^{o}}_{q,\tau }\right)[(\nu_{q,\xi^{o}}^{2}\|\omega_{q}-{℘}^{⋆}{\|}^{2}+\sigma_{\upsilon }\|\omega_{q}-\pi_{q,N}{\|}^{2}]\\ & - & {\beta^{o}}_{q,1}[\sum_{\xi^{o}=2}^{\upsilon }{\beta^{o}}_{q,\xi^{o}}\prod_{\tau =1}^{\xi^{o}-1}\left(1-{\beta^{o}}_{q,\tau }\right)\|\omega_{q}-\pi_{q,\xi^{o}-1}{\|}^{2}+\prod_{\tau =1}^{\upsilon }\left(1-{\beta^{o}}_{q,\tau }\right){\left\|\omega_{q}-\pi_{q,\upsilon }\right\|}^{2}]\\ & \leq & \left({\beta^{o}}_{q,1}+\sum_{\xi^{o}=2}^{\upsilon }{\beta^{o}}_{q,\xi^{o}}\prod_{\tau =1}^{\xi^{o}-1}\left(1-{\beta^{o}}_{q,\tau }\right)+\prod_{\tau =1}^{\xi^{o}-1}\left(1-{\beta^{o}}_{q,\tau }\right)\right){\left\|\omega_{q}-{℘}^{⋆}\right\|}^{2}\\ & + & \sum_{\xi^{o}=2}^{\upsilon }{\beta^{o}}_{q,\xi^{o}}\prod_{\tau =1}^{\xi^{o}-1}\left(1-{\beta^{o}}_{q,\tau }\right)(\left(\nu_{q,\varsigma }^{2}-1\right)\|\omega_{q}-{℘}^{⋆}{\|}^{2}+\prod_{\tau =1}^{\upsilon }\left(1-{\beta^{o}}_{q,\tau }\right)\left(\nu_{q,\xi^{o}}^{2}-1\right){\left\|\omega_{q}-{℘}^{⋆}\right\|}^{2}\\ & - & \left({\beta^{o}}_{q,1}-\sigma_{\xi^{o}-1}^{\left(1\right)}\right)\sum_{\xi^{o}=2}^{\upsilon }{\beta^{o}}_{q,\xi^{o}}\prod_{\tau =1}^{\xi^{o}-1}\left(1-{\beta^{o}}_{q,\tau }\right){\left\|\omega_{q}-\pi_{q,\xi^{o}-1}\right\|}^{2}\\ & - & \left({\beta^{o}}_{q,1}-\sigma_{\upsilon }^{\left(1\right)}\right)\prod_{\tau =1}^{\upsilon }\left(1-{\beta^{o}}_{q,\tau }\right){\left\|\omega_{q}-\pi_{q,\upsilon }\right\|}^{2} \end{array}$$

From Proposition 2.2, it follows, for *s* = 1, that

$$\begin{array}{ccc} \|s_{q}-{℘}^{⋆}{\|}^{2} & \leq & \|\omega_{q}-{℘}^{⋆}{\|}^{2}+\sum_{\xi^{o}=2}^{\upsilon }{\beta^{o}}_{q,\xi^{o}}\prod_{\tau =1}^{\xi^{o}-1}\left(1-{\beta^{o}}_{q,\tau }\right)(\left(\nu_{q,\xi^{o}}^{2}-1\right){\left\|\omega_{q}-{℘}^{⋆}\right\|}^{2}\\ & & +\prod_{\tau =1}^{\upsilon }\left(1-{\beta^{o}}_{q,\tau }\right)(\left(\nu_{q,\xi^{o}}^{2}-1\right){\left\|\omega_{q}-{℘}^{⋆}\right\|}^{2}\\ & & -[\sum_{\xi^{o}=2}^{\upsilon }{\beta^{o}}_{q,\xi^{o}}\prod_{\tau =1}^{\xi^{o}-1}\left(1-{\beta^{o}}_{q,\tau }\right)\left({\beta^{o}}_{q,1}-\sigma_{\xi^{o}-1}^{\left(1\right)}\right){\left\|\omega_{q}-\pi_{q,\xi^{o}-1}\right\|}^{2}\\ & & +\prod_{\tau =1}^{\upsilon }\left(1-{\beta^{o}}_{q,\tau }\right)({\beta^{o}}_{q,1}-\sigma_{q,\upsilon }^{\left(1\right)}{\left\|\omega_{q}-\pi_{q,\upsilon }\right\|}^{2}] \end{array}$$ (31)

Also, from (23) and Proposition 2.2 with *ω*_*q*_ = *η*, *u*_*q*_ = *t*, ℘^⋆^ = *u*, *s* = 1 and $\vartheta_{q,\xi^{o}}\in {ℑ}_{\xi^{o}}u_{q}$, it is not difficult to see (employing the same approach as in above) that

$$\begin{array}{ccc} \|w_{q}-{℘}^{⋆}{\|}^{2} & \leq & \zeta_{q,1}\|u_{q}-{℘}^{⋆}{\|}^{2}+\sum_{\xi^{o}=2}^{\upsilon }\zeta_{q,\xi^{o}}\prod_{\tau =1}^{\xi^{o}-1}\left(1-\zeta_{q,\tau }\right){\left\|u_{q}-{℘}^{⋆}\right\|}^{2}\\ & & +\prod_{\tau =1}^{\upsilon }\left(1-\zeta_{q,\tau }\right){\left\|u_{q}-{℘}^{⋆}\right\|}^{2}\\ & & -[\sum_{\xi^{o}=2}^{\upsilon }\zeta_{q,\xi^{o}}\prod_{\tau =1}^{\xi^{o}-1}\left(1-\zeta_{q,\tau }\right)\left(\zeta_{q,1}-\sigma_{\xi^{o}-1}^{\left(2\right)}\right){\left\|u_{q}-\pi_{q,\xi^{o}-1}\right\|}^{2}\\ & & +\prod_{\tau =1}^{\upsilon }\left(1-\zeta_{q,\tau }\right)\left(\zeta_{q,1}-\sigma_{q,\upsilon }^{\left(2\right)}\right){\left\|\omega_{q}-\pi_{q,\upsilon }\right\|}^{2}] \end{array}$$ (32)

From (31) and (32), we obtain

$$\begin{array}{ccc} \|s_{q}-{℘}^{⋆}{\|}^{2} & \leq & [1+\sum_{\xi^{o}=2}^{\upsilon }{\beta^{o}}_{q,\xi^{o}}\prod_{\tau =1}^{\xi^{o}-1}\left(1-{\beta^{o}}_{q,\tau }\right)\left(\nu_{q}^{2}-1\right)+\prod_{\tau =1}^{\upsilon }\left(1-{\beta^{o}}_{q,\tau }\right)\left(\nu_{q}^{2}-1\right)]\{[\zeta_{q,1}\\ & & +\sum_{\xi^{o}=2}^{\upsilon }\zeta_{q,\xi^{o}}\prod_{\tau =1}^{\xi^{o}-1}\left(1-\zeta_{q,\tau }\right)+\prod_{\tau =1}^{\upsilon }\left(1-\zeta_{q,\tau }\right)]\|{℘}_{q}-{℘}^{⋆}{\|}^{2}\\ & & -[\sum_{\xi^{o}=2}^{\upsilon }\zeta_{q,\xi^{o}}\prod_{\tau =1}^{\xi^{o}-1}\left(1-\zeta_{q,\tau }\right)\left(\zeta_{q,1}-\sigma_{\xi^{o}-1}^{\left(2\right)}\right){\left\|u_{q}-\vartheta_{q,\xi^{o}-1}\right\|}^{2}\\ & & +\prod_{j=1}^{\upsilon }\left(1-\zeta_{q,\tau }\right)\left(\zeta_{q,1}-\sigma_{\upsilon }^{\left(2\right)}\right){\left\|\omega_{q}-\vartheta_{q,\upsilon }\right\|}^{2}]\}\\ & & -[\sum_{\xi^{o}=2}^{\upsilon }{\beta^{o}}_{q,\xi^{o}}\prod_{\tau =1}^{\xi^{o}-1}\left(1-{\beta^{o}}_{q,\tau }\right)\left({\beta^{o}}_{q,1}-\sigma_{\xi^{o}-1}^{\left(1\right)}\right){\left\|\omega_{q}-\pi_{q,\xi^{o}-1}\right\|}^{2}\\ & & +\prod_{\tau =1}^{\upsilon }\left(1-{\beta^{o}}_{q,\tau }\right)\left({\beta^{o}}_{q,1}-\sigma_{\upsilon }^{\left(1\right)}\right){\left\|\omega_{q}-\pi_{q,\upsilon }\right\|}^{2}]\\ & \leq & \{\zeta_{q,1}+\sum_{\xi^{o}=2}^{\upsilon }\zeta_{q,\xi^{o}}\prod_{\tau =1}^{\xi^{o}-1}\left(1-\zeta_{q,\tau }\right)+\prod_{\tau =1}^{\upsilon }\left(1-\zeta_{q,j}\right)\\ & & +(\sum_{\xi^{o}=2}^{\upsilon }{\beta^{o}}_{q,\xi^{o}}\prod_{\tau =1}^{\xi^{o}-1}\left(1-{\beta^{o}}_{q,\tau }\right)+\prod_{\tau =1}^{\upsilon }\left(1-{\beta^{o}}_{q,\tau }\right))[\zeta_{q,1}+\sum_{\xi^{o}=2}^{\upsilon }\zeta_{q,\xi^{o}}\prod_{\tau =1}^{\xi^{o}-1}\left(1-\zeta_{q,\tau }\right)\\ & & +\prod_{\tau =1}^{\upsilon }\left(1-\zeta_{q,\tau }\right)]\left(\nu_{q}^{2}-1\right)\}{\left\|{℘}_{q}-{℘}^{⋆}\right\|}^{2}\\ & & -[\sum_{\xi^{o}=2}^{\upsilon }\zeta_{q,\xi^{o}}\prod_{\tau =1}^{\xi^{o}-1}\left(1-\zeta_{q,\tau }\right)\left(\zeta_{q,1}-\sigma_{\xi^{o}-1}^{\left(2\right)}\right){\left\|u_{q}-\vartheta_{q,\xi^{o}-1}\right\|}^{2}\\ & & +\prod_{\tau =1}^{\upsilon }\left(1-\zeta_{q,\tau }\right)\left(\zeta_{q,1}-\sigma_{\upsilon }^{\left(2\right)}\right){\left\|\omega_{q}-\vartheta_{q,\upsilon }\right\|}^{2}]\\ & & -[\sum_{\xi^{o}=2}^{\upsilon }{\beta^{o}}_{q,\xi^{o}}\prod_{j=1}^{\xi^{o}-1}\left(1-{\beta^{o}}_{q,\tau }\right)\left({\beta^{o}}_{q,1}-\sigma_{\xi^{o}-1}^{\left(1\right)}\right){\left\|\omega_{q}-\pi_{q,\xi^{o}-1}\right\|}^{2} \end{array}$$ (33)

$$\begin{array}{ccc} & & +\prod_{\tau =1}^{\upsilon }\left(1-{\beta^{o}}_{q,\tau }\right)\left({\beta^{o}}_{q,1}-\sigma_{\upsilon }^{\left(1\right)}\right){\left\|\omega_{q}-\pi_{q,N}\right\|}^{2}] \end{array}$$ (34)

Also, using (23), we obtain the following estimates:

$$\begin{array}{ccc} \|{℘}_{q+1}-{℘}^{⋆}\| & \leq & \|\alpha_{n}\gamma f\left({℘}_{q}\right)+\delta_{q}s_{q}+\left(\left(1-\delta_{q}\right)I-\alpha_{q}A\right)s_{q}-{℘}^{⋆}\|\\ & \leq & \alpha_{q}\|\gamma f\left({℘}_{q}\right)-A{℘}^{⋆}\|+\delta_{q}\|s_{q}-{℘}^{⋆}\left\|+\right\|\left(1-\delta_{q}\right)I-\alpha_{q}A\|\|s_{q}-{℘}^{⋆}\|\\ & \leq & \alpha_{q}\gamma \|f\left({℘}_{q}\right)-f\left({℘}^{⋆}\right)\|+\alpha_{q}\|\gamma f\left({℘}^{⋆}\right)-A{℘}^{⋆}\|+\delta_{q}\left\|s_{q}-{℘}^{⋆}\right\|\\ & & +\left(\left(1-\delta_{q}\right)I-\alpha_{n}\bar{\alpha }\right)\left\|s_{q}-{℘}^{⋆}\right\|\\ & \leq & \alpha_{q}\gamma \rho \|{℘}_{n}-{℘}^{⋆}\|+\alpha_{q}\|\gamma f\left({℘}^{⋆}\right)-A{℘}^{⋆}\|+\left(1-\alpha_{q}\bar{\alpha }\right)\left\|s_{q}-{℘}^{⋆}\right\| \end{array}$$ (35)

By applying conditions [(i) and (iv)] in (33), we get

$$\begin{array}{ccc} \|s_{q}-{℘}^{⋆}\| & \leq & [1+\sum_{\xi^{o}=2}^{\upsilon }{\beta^{o}}_{q,\xi^{o}}\prod_{\tau =1}^{\xi^{o}-1}\left(1-{\beta^{o}}_{q,\tau }\right)\left(\nu_{q}^{2}-1\right)+\prod_{\tau =1}^{\xi^{o}-1}\left(1-{\beta^{o}}_{q,\tau }\right)\left(\nu_{q}^{2}-1\right)]\left\|{℘}_{q}-{℘}^{⋆}\right\|\\ & \leq & \left(1+2\epsilon \alpha_{q}\right)\left\|{℘}_{q}-{℘}^{⋆}\right\| \end{array}$$ (36)

From (35) and (36), we obtain

$$\begin{array}{ccc} \|{℘}_{q+1}-{℘}^{⋆}\| & \leq & \alpha_{q}\gamma \rho \|{℘}_{q}-{℘}^{⋆}\|+\alpha_{q}\|\gamma f\left({℘}^{⋆}\right)-A{℘}^{⋆}\|+\left(1-\alpha_{q}\bar{\alpha }\right)\left(1+2\epsilon \alpha_{q}\right)\left\|{℘}_{q}-{℘}^{⋆}\right\|\\ & \leq & \alpha_{q}\gamma \rho \|{℘}_{q}-{℘}^{⋆}\|+\alpha_{q}\|\gamma f\left({℘}^{⋆}\right)-A{℘}^{⋆}\|+\left(1+2\epsilon \alpha_{q}-\alpha_{n}\bar{\alpha }\right)\left\|{℘}_{q}-{℘}^{⋆}\right\|\\ & = & \left[1-\left(\bar{\alpha }-2\epsilon -\gamma \rho \right)\alpha_{q}\right]\|{℘}_{q}-{℘}^{⋆}\|+\alpha_{q}\left\|\gamma f\left({℘}^{⋆}\right)-A{℘}^{⋆}\right\| \end{array}$$

Employing mathematical inductional argument, we have

$$\begin{array}{c} \|{℘}_{q}-{℘}^{⋆}\|\leq \text{max}\{\|{℘}_{0}-{℘}^{⋆}\|,\frac{\|\gamma f\left({℘}^{⋆}\right)-A{℘}^{⋆}\|}{\bar{\alpha }-2\epsilon -\gamma \rho }\},\forall q\in N. \end{array}$$

The last inequality implies that the sequence ${\left\{{℘}_{q}\right\}}_{q=0}^{\infty }$ is bounded; and as a consequence, the boundedness of the following sequences: ${\left\{u_{q}\right\}}_{n=0}^{\infty },{\left\{s_{q}\right\}}_{q=0}^{\infty }$ and ${\left\{f\left({℘}_{q}\right)\right\}}_{q=0}^{\infty }$ are assured.

Next, for each *i*, we prove the following conclusion: ‖*ω*_*n*_ − *π*_*n*,*i*_‖ → 0 and $\|u_{q}-\vartheta_{q,\varsigma }\|\to 0$ as *q* → ∞. Using Lemma 2.1, (34) and (36), we get the following estimates:

$$\begin{array}{ccc} \|{℘}_{q+1}-{℘}^{⋆}{\|}^{2} & \leq & \|\alpha_{q}\gamma f\left({℘}_{q}\right)+\delta_{q}s_{q}+\left(\left(1-\delta_{q}\right)I-\alpha_{q}A\right)s_{q}-{℘}^{⋆}{\|}^{2}\\ & \leq & \|\alpha_{q}\left(\gamma f\left({℘}_{q}\right)-A{℘}^{⋆}\right)-\delta_{q}\left({℘}^{⋆}-s_{q}\right)+\left(\left(1-\delta_{q}\right)I-\alpha_{q}A\right)\left(s_{q}-{℘}^{⋆}\right){\|}^{2}\\ & = & \|\alpha_{q}\left(\gamma f\left({℘}_{q}\right)-A{℘}^{⋆}\right)-\delta_{q}\left({℘}^{⋆}-s_{q}\right){\|}^{2}+{\left\|\left(\left(1-\delta_{q}\right)I-\alpha_{q}A\right)\left(s_{q}-{℘}^{⋆}\right)\right\|}^{2}\\ & & +2\alpha_{q}\|\gamma f\left({℘}_{q}\right)-A{℘}^{⋆}\left\|\right\|\left(\left(1-\delta_{q}\right)I-\alpha_{q}A\right)\left(s_{q}-{℘}^{⋆}\right)\|\\ & \leq & \alpha_{q}^{2}\|\gamma f\left({℘}_{q}\right)-A{℘}^{⋆}{\|}^{2}+\delta_{q}^{2}\|{℘}^{⋆}-s_{n}{\|}^{2}+{\left(\left(1-\delta_{q}\right)I-\alpha_{q}\bar{\alpha }\right)}^{2}{\left\|s_{n}-{℘}^{⋆}\right\|}^{2}\\ & & +2\alpha_{q}\left(\left(1-\delta_{q}\right)I-\alpha_{q}\bar{\alpha }\right)\|\gamma f\left({℘}_{q}\right)-A{℘}^{⋆}\left\|\right\|s_{q}-q\|\\ & \leq & \left(1-2\delta_{q}+\delta_{q}^{2}-2\left(1-\delta_{q}\right)\alpha_{q}\bar{\alpha }+\alpha_{q}^{2}{\bar{\alpha }}^{2}\right)\|s_{q}-{℘}^{⋆}{\|}^{2}+\delta_{q}^{2}{\left\|{℘}^{⋆}-s_{q}\right\|}^{2}\\ & & +\alpha_{q}^{2}\|\gamma f\left({℘}_{q}\right)-A{℘}^{⋆}{\|}^{2}+2\alpha_{q}\left(\left(1-\delta_{q}\right)I-\alpha_{q}\bar{\alpha }\right)\|\gamma f\left({℘}_{q}\right)-A{℘}^{⋆}\left\|\right\|s_{q}-{℘}^{⋆}\|\\ & = & \left(1-2\delta_{q}\left(\delta_{q}-1\right)+\alpha_{q}\bar{\alpha }\left(\alpha_{qn}\alpha -2\right)+2\delta_{q}\alpha_{q}\bar{\alpha }\right){\left\|s_{q}-{℘}^{⋆}\right\|}^{2}\\ & & +\alpha_{q}^{2}\|\gamma f\left({℘}_{q}\right){-Aq\|}^{2}+2\alpha_{q}\left(\left(1-\delta_{q}\right)I-\alpha_{q}\bar{\alpha }\right)\|\gamma f\left({℘}_{q}\right)-A{℘}^{⋆}\left\|\right\|s_{q}-{℘}^{⋆}\|\\ & \leq & \left(1+2\delta_{q}\alpha_{q}\bar{\alpha }\right)\|s_{q}-{℘}^{⋆}{\|}^{2}+\alpha_{q}^{2}{\left\|\gamma f\left({℘}_{q}\right)-{℘}^{⋆}\right\|}^{2}\\ & & +2\alpha_{q}\left(\left(1-\delta_{q}\right)I-\alpha_{q}\bar{\alpha }\right)\|\gamma f({℘}_{q}-{℘}^{⋆}\left\|\right\|s_{q}-{℘}^{⋆}\| \end{array}$$ (37)

$$\begin{array}{ccc} & \leq & \{\zeta_{q,1}+\sum_{\xi^{o}=2}^{\upsilon }\zeta_{q,\xi^{o}}\prod_{\tau =1}^{\xi^{o}-1}\left(1-\zeta_{q,\tau }\right)+\prod_{\tau =1}^{\upsilon }\left(1-\zeta_{q,j}\right)\\ & + & (\sum_{\xi^{o}=2}^{\upsilon }{\beta^{o}}_{q,\xi^{o}}\prod_{\tau =1}^{\xi^{o}-1}\left(1-{\beta^{o}}_{q,\tau }\right)\\ & + & \prod_{\tau =1}^{\upsilon }\left(1-{\beta^{o}}_{q,\tau }\right))[\zeta_{q,1}+(\sum_{\xi^{o}=2}^{\upsilon }\zeta_{q,\xi^{o}}\prod_{\tau =1}^{\xi^{o}-1}\left(1-\zeta_{q,\tau }\right)\\ & + & \prod_{\tau =1}^{\upsilon }\left(1-\zeta_{q,\tau }\right))]\left(\nu_{q}^{2}-1\right)\}{\left\|{℘}_{q}-{℘}^{⋆}\right\|}^{2} \end{array}$$ (38)

$$\begin{array}{ccc} & - & [\sum_{\xi^{o}=2}^{\upsilon }\zeta_{q,\xi^{o}}\prod_{\tau =1}^{\xi^{o}-1}\left(1-\zeta_{q,\tau }\right)\left(\zeta_{q,1}-\sigma_{\xi^{o}-1}^{\left(2\right)}\right){\left\|u_{q}-\vartheta_{q,\xi^{o}-1}\right\|}^{2}\\ & & +\prod_{\tau =1}^{\upsilon }\left(1-\zeta_{n,j}\right)\left(\zeta_{q,1}-\sigma_{\upsilon }^{\left(2\right)}\right){\left\|u_{q}-\vartheta_{q,\upsilon }\right\|}^{2}]\\ & & -[\sum_{\xi^{o}=2}^{\upsilon }{\beta^{o}}_{q,\xi^{o}}\prod_{\tau =1}^{\xi^{o}-1}\left(1-{\beta^{o}}_{q,\tau }\right)\left({\beta^{o}}_{q,1}-\sigma_{\xi^{o}-1}^{\left(1\right)}\right){\left\|\omega_{q}-\pi_{q,\xi^{o}-1}\right\|}^{2}\\ & & +\prod_{\tau =1}^{\upsilon }\left(1-{\beta^{o}}_{q,\tau }\right)\left({\beta^{o}}_{q,1}-\sigma_{\upsilon }^{\left(1\right)}\right){\left\|\omega_{q}-\pi_{q,\upsilon }\right\|}^{2}]\\ & & +2\delta_{q}\alpha_{q}\bar{\alpha }\{\zeta_{q,1}+\sum_{\xi^{o}=2}^{\upsilon }\zeta_{q,\xi^{o}}\prod_{\tau =1}^{\xi^{o}-1}\left(1-\zeta_{q,\tau }\right)+\prod_{\tau =1}^{\upsilon }\left(1-\zeta_{q,\tau }\right)\\ & & +(\sum_{\xi^{o}=2}^{\upsilon }{\beta^{o}}_{q,\xi^{o}}\prod_{\tau =1}^{\xi^{o}-1}\left(1-{\beta^{o}}_{q,\tau }\right)+\prod_{\tau =1}^{\upsilon }\left(1-{\beta^{o}}_{q,\tau }\right))[\zeta_{q,1}+(\sum_{\xi^{o}=2}^{\upsilon }\zeta_{q,\xi^{o}}\prod_{\tau =1}^{\xi^{o}-1}\left(1-\zeta_{q,\tau }\right)\\ & & +\prod_{\tau =1}^{\upsilon }\left(1-\zeta_{q,j}\right))]\left(\nu_{q}^{2}-1\right)\}{\left\|{℘}_{q}-q\right\|}^{2}-[\sum_{\xi^{o}=2}^{\upsilon }\zeta_{q,\xi^{o}}\prod_{\tau =1}^{\xi^{o}-1}\left(1-\zeta_{q,\tau }\right)\\ & & \times \left(\zeta_{q,1}-\sigma_{\xi^{o}-1}^{\left(2\right)}\right){\left\|u_{q}-\vartheta_{q,\xi^{o}-1}\right\|}^{2}\\ & & +\prod_{\tau =1}^{\upsilon }\left(1-\zeta_{q,j}\right)\left(\zeta_{q,1}-\sigma_{\upsilon }^{\left(2\right)}\right){\left\|\omega_{q}-\vartheta_{q,\upsilon }\right\|}^{2}]\\ & & -[\sum_{\xi^{o}=2}^{\upsilon }{\beta^{o}}_{q,\xi^{o}}\prod_{\tau =1}^{\xi^{o}-1}\left(1-{\beta^{o}}_{q,\tau }\right)\left({\beta^{o}}_{q,1}-\sigma_{\xi^{o}-1}^{\left(1\right)}\right){\left\|\omega_{q}-\pi_{q,\xi^{o}-1}\right\|}^{2}\\ & & +\prod_{\tau =1}^{\upsilon }\left(1-{\beta^{o}}_{q,\tau }\right)\left({\beta^{o}}_{q,1}-\sigma_{\upsilon }^{\left(1\right)}\right)\|\omega_{q}-\pi_{q,\upsilon }{\|}^{2}]+\alpha_{q}^{2}{\left\|\zeta f\left({℘}_{q}\right)-A{℘}^{⋆}\right\|}^{2}\\ & & +2\alpha_{q}\left(1+2\epsilon \alpha_{q}\right)\left(\left(1-\delta_{q}\right)I-\alpha_{q}\bar{\alpha }\right)\|\gamma f\left({℘}_{q}\right)-Aq\|\|{℘}_{q}-{℘}^{⋆}\|. \end{array}$$ (39)

By setting

$$\begin{array}{ccc} ℜ & = & \sum_{\xi^{o}=2}^{\upsilon }\zeta_{q,\xi^{o}}\prod_{\tau =1}^{\xi^{o}-1}\left(1-\zeta_{q,\tau }\right)\left(\gamma_{q,1}-\sigma_{\varsigma -1}^{\left(2\right)}\right){\left\|u_{q}-\vartheta_{q,\varsigma -1}\right\|}^{2}\\ & + & \prod_{\tau =1}^{\xi^{o}-1}\left(1-\zeta_{q,\tau }\right)\left(\zeta_{q,1}-\sigma_{\upsilon }^{\left(2\right)}\right){\left\|u_{q}-\vartheta_{q,\upsilon }\right\|}^{2}, \end{array}$$

The inequality above becomes

$$\begin{array}{ccc} ℜ & \leq & \|{℘}_{q}-{℘}^{⋆}{\|}^{2}-{\left\|{℘}_{q+1}-{℘}^{⋆}\right\|}^{2}+\{\{(\sum_{\xi^{o}=2}^{\upsilon }\zeta_{q,\xi^{o}}\prod_{\tau =1}^{\xi^{o}-1}\left(1-\zeta_{q,\tau }\right)+\prod_{\tau =1}^{\upsilon }\left(1-\zeta_{q,\tau }\right))\\ & & +(\sum_{\xi^{o}=2}^{\upsilon }{\beta^{o}}_{q,\xi^{o}}\prod_{\tau =1}^{\xi^{o}-1}\left(1-{\beta^{o}}_{q,\tau }\right)+\prod_{\tau =1}^{\upsilon }\left(1-{\beta^{o}}_{q,\tau }\right))[\zeta_{q,1}+(\sum_{\xi^{o}=2}^{\upsilon }\zeta_{q,\xi^{o}}\prod_{\tau =1}^{\xi^{o}-1}\left(1-\zeta_{q,\tau }\right)\\ & & +\prod_{\tau =1}^{\upsilon }\left(1-\zeta_{q,\tau }\right))]\left(\nu_{q}^{2}-1\right)\}\|{℘}_{q}-{℘}^{⋆}{\|}^{2}-[\sum_{\xi^{o}=2}^{\upsilon }{\beta^{o}}_{q,\xi^{o}}\prod_{\tau =1}^{\xi^{o}-1}\left(1-{\beta^{o}}_{q,\tau }\right)\left({\beta^{o}}_{q,1}-\sigma_{i-1}^{\left(1\right)}\right){\left\|\omega_{n}-\pi_{n,i-1}\right\|}^{2}\\ & & +\prod_{\tau =1}^{\upsilon }\left(1-{\beta^{o}}_{q,\tau }\right)\left({\beta^{o}}_{q,1}-\sigma_{q,\upsilon }^{\left(1\right)}\right){\left\|\omega_{q}-\pi_{q,\upsilon }\right\|}^{2}]\\ & & +2\delta_{q}\alpha_{q}\bar{\alpha }\{\zeta_{q,1}+(\sum_{\xi^{o}=2}^{\upsilon }\zeta_{q,\xi^{o}}\prod_{\tau =1}^{\xi^{o}-1}\left(1-\zeta_{q,\tau }\right)+\prod_{\tau =1}^{\upsilon }\left(1-\zeta_{q,\tau }\right))\\ & & +(\sum_{\xi^{o}=2}^{\upsilon }{\beta^{o}}_{q,\xi^{o}}\prod_{\tau =1}^{\xi^{o}-1}\left(1-{\beta^{o}}_{q,\tau }\right)+\prod_{\tau =1}^{\upsilon }\left(1-{\beta^{o}}_{q,\tau }\right))[\zeta_{q,1}+(\sum_{\xi^{o}=2}^{\upsilon }\zeta_{q,\xi^{o}}\prod_{\tau =1}^{\xi^{o}-1}\left(1-\zeta_{q,\tau }\right)\\ & & +\prod_{\tau =1}^{\upsilon }\left(1-\zeta_{q,\tau }\right))]\left(\nu_{q}^{2}-1\right)\}{\left\|{℘}_{q}-{℘}^{⋆}\right\|}^{2}\\ & & -[\sum_{\xi^{o}=2}^{\upsilon }{\beta^{o}}_{q,\xi^{o}}\prod_{\tau =1}^{\xi^{o}-1}\left(1-{\beta^{o}}_{q,\tau }\right)\left({\beta^{o}}_{n,1}-\sigma_{\xi^{o}-1}^{\left(1\right)}\right){\left\|\omega_{q}-\pi_{q,\xi^{o}-1}\right\|}^{2}\\ & & +\prod_{\tau =1}^{\upsilon }\left(1-{\beta^{o}}_{q,\tau }\right)\left({\beta^{o}}_{q,1}-\sigma_{q,\upsilon }^{\left(1\right)}\right)\|\omega_{q}-\pi_{q,\upsilon }{\|}^{2}]\}+\alpha_{q}^{2}{\left\|\gamma f\left({℘}_{q}\right)-A{℘}^{⋆}\right\|}^{2}\\ & & +2\alpha_{q}\left(1+2\epsilon \alpha_{q}\right)\left(\left(1-\delta_{q}\right)I-\alpha_{q}\bar{\alpha }\right)\|\gamma f\left({℘}_{q}\right)-A{℘}^{⋆}\left\|\right\|{℘}_{q}-{℘}^{⋆}\|. \end{array}$$ (40)

To established that ℘_*q*_ → ℘^⋆^ as *q* → ∞, consider the two Cases below:

Case A: Let $\left\{\right\|{℘}_{q}-{℘}^{⋆}{\left\|\right\}}_{q=0}^{\infty }$ be monotonically decreasing. Then, $\left\{\right\|{℘}_{q}-{℘}^{⋆}{\left\|\right\}}_{q=0}^{\infty }$ is convergent. Therefore,

$$\begin{array}{c} \underset{q\to \infty }{\text{lim}}\left[\right\|{℘}_{q}-{℘}^{⋆}\left\|-\right\|{℘}_{q+1}-{℘}^{⋆}\left\|\right]=0. \end{array}$$ (41)

Hence, (40) and (41) in company with (i), (ii), (iv) and the characteristic property of {*ν*_*q*_} give

$$\begin{array}{c} \underset{q\to \infty }{\text{lim}}\left\|u_{q}-\vartheta_{n,i}\right\|=0. \end{array}$$ (42)

Since $\vartheta_{q,\varsigma }\in {ℑ}_{\varsigma }u_{q},$ it follows from (42) that

$$\begin{array}{c} \underset{q\to \infty }{\text{lim}}\left\|u_{q}-{ℑ}_{\varsigma }u_{q}\right\|=0. \end{array}$$ (43)

Applying the same line of thought as in above (taking into account (40) and (41), (i), (iii), (iv) and the characteristic property of {*ν*_*q*_}), it will not be difficult to see that

$$\begin{array}{c} \underset{q\to \infty }{\text{lim}}\left\|\omega_{q}-\pi_{q,\varsigma }\right\|=0. \end{array}$$ (44)

Since $\pi_{q,i}\in {ð}_{\varsigma }^{q}\omega_{q},$ employing (44) we have

$$\begin{array}{c} \|\omega_{q}-{ð}_{\varsigma }^{q}\omega_{q}\|=0. \end{array}$$ (45)

Next, we prove that $\underset{q\to \infty }{\text{lim}}\left\|{℘}_{q}-u_{q}\right\|=0$. For any ${℘}^{⋆}\in F,$ we get

$$\begin{array}{ccc} \|u_{q}-{℘}^{⋆}{\|}^{2} & \leq & \|Tr_{q}{℘}_{q}-Tr_{q}{℘}^{⋆}{\|}^{2}\\ & \leq & \left\langle Tr_{q}{℘}_{q}-Tr_{q}{℘}^{⋆},{℘}_{q}-{℘}^{⋆}\right\rangle \\ & = & \left\langle u_{q}-{℘}^{⋆},{℘}_{q}-{℘}^{⋆}\right\rangle \\ & = & \frac{1}{2}(\|u_{q}-{℘}^{⋆}{\|}^{2}+\|{℘}_{q}-{℘}^{⋆}{\|}^{2}-{\left\|{℘}_{q}-u_{q}\right\|}^{2}) \end{array}$$

so that

$$\begin{array}{c} \|u_{q}-{℘}^{⋆}{\|}^{2}\leq \|{℘}_{q}-{℘}^{⋆}{\|}^{2}-{\left\|{℘}_{q}-u_{q}\right\|}^{2}. \end{array}$$ (46)

From (31), (32) and (36), we have

$$\begin{array}{ccc} \|{℘}_{q+1}-{℘}^{⋆}{\|}^{2} & \leq & \|\omega_{q}-{℘}^{⋆}{\|}^{2}+\sum_{\xi^{o}=2}^{\upsilon }{\beta^{o}}_{q,\xi^{o}}\prod_{\tau =1}^{\xi^{o}-1}\left(1-{\beta^{o}}_{q,\tau }\right)\left(\nu_{q}^{2}-1\right){\left\|\omega_{q}-{℘}^{⋆}\right\|}^{2}\\ & & +\prod_{\tau =1}^{\upsilon }\left(1-{\beta^{o}}_{q,\tau }\right)\left(\nu_{q}^{2}-1\right)\|\omega_{q}-{℘}^{⋆}{\|}^{2}+2\alpha_{q}[\delta_{q}\bar{\alpha }{\left\|s_{q}-{℘}^{⋆}\right\|}^{2}\\ & & +\left(\left(1-\delta_{q}\right)I-\alpha_{q}\bar{\alpha }\right)\|\gamma f\left({℘}_{q}\right)-A{℘}^{⋆}\left\|\right\|s_{n}-{℘}^{⋆}\left\|\right]\\ & \leq & \|u_{q}-{℘}^{⋆}{\|}^{2}+\sum_{\xi^{o}=2}^{\upsilon }\zeta_{q,\xi^{o}}\prod_{\tau =1}^{\xi^{o}-1}\left(1-\zeta_{q,\tau }\right)\left(\nu_{q}^{2}-1\right){\left\|u_{q}-{℘}^{⋆}\right\|}^{2}\\ & & +\prod_{\tau =1}^{\upsilon }\left(1-\gamma_{q,\tau }\right)\left(\nu_{q}^{2}-1\right){\left\|u_{q}-{℘}^{⋆}\right\|}^{2}\\ & & +\sum_{\xi^{o}=2}^{\upsilon }{\beta^{o}}_{q,\xi^{o}}\prod_{\tau =1}^{\xi^{o}-1}\left(1-{\beta^{o}}_{q,\tau }\right)\left(\nu_{q}-1\right){\left\|\omega_{q}-{℘}^{⋆}\right\|}^{2}\\ & & +\prod_{\tau =1}^{\upsilon }\left(1-{\beta^{o}}_{q,\tau }\right)\left(\nu_{q}^{2}-1\right)\|\omega_{q}{-q\|}^{2}+2\alpha_{q}[\delta_{q}\bar{\alpha }{\left\|s_{q}-{℘}^{⋆}\right\|}^{2}\\ & & +\left(\left(1-\delta_{q}\right)I-\alpha_{q}\bar{\alpha }\right)\|\gamma g\left({℘}_{q}\right)-A{℘}^{⋆}\left\|\right\|s_{q}-{℘}^{⋆}\left\|\right], \end{array}$$

which by (46) gives

$$\begin{array}{ccc} \|{℘}_{q}-u_{q}{\|}^{2} & \leq & \|{℘}_{q}-{℘}^{⋆}{\|}^{2}-{\left\|{℘}_{q+1}-{℘}^{⋆}\right\|}^{2}+[\sum_{\xi^{o}=2}^{\upsilon }\zeta_{q,\xi^{o}}\prod_{\tau =1}^{\xi^{o}-1}\left(1-\zeta_{q,\tau }\right)\\ & & +\prod_{\tau =1}^{\upsilon }\left(1-\zeta_{q,\tau }\right)+\sum_{\xi^{o}=2}^{\upsilon }{\beta^{o}}_{q,\xi^{o}}\prod_{\tau =1}^{\xi^{o}-1}\left(1-{\beta^{o}}_{q,\tau }\right)\left(\nu_{q}^{2}-1\right)\\ & & +\prod_{\tau =1}^{\upsilon }\left(1-{\beta^{o}}_{q,\tau }\right)\left(\nu_{q}^{2}-1\right)]\varpi +2\alpha_{q}[\delta_{q}\bar{\alpha }\varpi \\ & & +\left(\left(1-\delta_{q}\right)I-\alpha_{q}\bar{\alpha }\right)\|\gamma f\left({℘}_{q}\right)-A{℘}^{⋆}\left\|\right\|s_{q}-{℘}^{⋆}\left\|\right] \end{array}$$ (47)

where $\varpi =\text{sup}\|u_{q}-{℘}^{⋆}{\|}^{2}$,$\varpi^{⋆⋆}=\text{sup}{\left\|\omega_{q}-{℘}^{⋆}\right\|}^{2}$ and $\varpi =max\{\varpi^{⋆},\varpi^{⋆⋆}\}$.

Using (41), condition (iv) and the characterization of {*ν*_*q*_}, we get from the last inequality that

$$\begin{array}{c} \underset{q\to \infty }{\text{lim}}\left\|{℘}_{q}-u_{q}\right\|=0. \end{array}$$ (48)

Furthermore, the following estimates are due to the application of (23) and Proposition 2.2:

$$\begin{array}{ccc} \|{℘}_{q+1}-s_{q}\| & \leq & \|\alpha_{q}\alpha f\left({℘}_{q}\right)+\delta_{q}s_{q}+\left(\left(1-\delta_{q}\right)I-A\right)s_{q}-s_{q}\|\\ & = & \alpha_{q}\left\|\alpha f\left({℘}_{q}\right)-As_{q}\right\|\to 0\;as\;q\to \infty \;\left(by\;condition\;\left(iv\right)\right), \end{array}$$ (49)

$$\begin{array}{ccc} \|s_{q}-\omega_{q}{\|}^{2} & \leq & \sum_{\xi^{o}=2}^{\upsilon }{\beta^{o}}_{q,\xi^{o}}\prod_{\tau =1}^{\xi^{o}-1}\left(1-{\beta^{o}}_{q,\tau }\right){\left\|\pi_{q,\varsigma -1}-\omega_{q}\right\|}^{2}\\ & & +\prod_{\tau =1}^{\upsilon }\left(1-{\beta^{o}}_{q,j}\right){\left\|\pi_{q,N}-\omega_{q}\right\|}^{2}\to 0\;as\;q\to \infty \;\left[by\;\left(44\right)\right], \end{array}$$ (50)

and by using (23), Proposition 2.2 and (42), we have

$$\begin{array}{ccc} \|\omega_{q}-u_{q}{\|}^{2} & \leq & \sum_{\xi^{o}=2}^{\upsilon }\zeta_{q,\xi^{o}}\prod_{\tau =1}^{\xi^{o}-1}\left(1-\zeta_{q,\tau }\right){\left\|\vartheta_{q,\varsigma -1}-u_{q}\right\|}^{2}\\ & & +\prod_{\tau =1}^{\upsilon }\left(1-\zeta_{q,j}\right){\left\|\nu_{q,\upsilon }-u_{q}\right\|}^{2}\to 0\;as\;q\to \infty . \end{array}$$ (51)

Now, observe that

$$\begin{array}{c} \|{℘}_{q+1}-{℘}_{q}\left\|\leq \right\|{℘}_{q+1}-s_{q}\left\|+\right\|s_{q}-\omega_{q}\left\|+\right\|\omega_{q}-u_{q}\left\|+\right\|u_{q}-{℘}_{q}\|, \end{array}$$

which by (48), (49), (50) and (51) yields

$$\begin{array}{c} \underset{q\to \infty }{\text{lim}}\left\|{℘}_{q+1}-{℘}_{q}\right\|=0. \end{array}$$ (52)

Also, observe that

$$\begin{array}{ccc} \|\omega_{q}-{ð}_{\varsigma }\omega_{q}\| & \leq & \|\omega_{q}-{ð}_{i}^{q}\omega_{q}\left\|+\right\|{ð}_{\varsigma }^{q}\omega_{q}-{ð}_{\varsigma }^{q}u_{q}\left\|+\right\|{ð}_{\varsigma }\left({ð}_{\varsigma }^{q-1}u_{q}\right)-{ð}_{\varsigma }\left({ð}^{q-1}\omega_{q-1}\right)\|\\ & & +\|{ð}_{\varsigma }\left({ð}_{\varsigma }^{q-1}\omega_{q-1}\right)-{ð}_{\varsigma }\omega_{q})\|\\ & \leq & \|\omega_{q}-{ð}_{\varsigma }^{q}\omega_{q}\left\|+L\right\|\omega_{q}-u_{q}\left\|+L\right\|u_{q}-\omega_{q-1}\left\|+L\right\|{ð}_{\varsigma }^{q-1}\omega_{q-1}-\omega_{q}\|\\ & \leq & \|\omega_{q}-{ð}_{\varsigma }^{q}\omega_{q}\left\|+L\right\|\omega_{q}-u_{q}\left\|+L[\right\|u_{q}-{℘}_{q}\left\|+\right\|{℘}_{q}-s_{q-1}\left\|+\right\|s_{q-1}-\omega_{q-1}\left\|\right]\\ & & +L[\|{ð}_{\varsigma }^{q-1}\omega_{q-1}-\omega_{n-1}\|+\|\omega_{q-1}-s_{n-1}\|+\|s_{q-1}-{℘}_{q}\|+\|{℘}_{q}-u_{q}\|\\ & & +\|u_{q}-\omega_{q}\left\|\right]\\ & \leq & \|\omega_{q}-{ð}_{\varsigma }^{q}\omega_{q}\left\|+L\right\|{ð}_{\varsigma }^{q-1}\omega_{q-1}-\omega_{q-1}\left\|+2L[\right\|\omega_{q}-u_{q}\left\|+\right\|u_{q}-{℘}_{q}\|\\ & & +\|{℘}_{q}-s_{q-1}\left\|+\right\|s_{q-1}-\omega_{q-1}\left\|\right], \end{array}$$ (53)

which, from (45), (48), (49), (50) and (51), we obtain

$$\begin{array}{c} \underset{q\to \infty }{\text{lim}}\left\|\omega_{q}-G_{\varsigma }\omega_{q}\right\|=0. \end{array}$$ (54)

Next, we prove that

$$\begin{array}{c} \underset{q\to \infty }{\text{lim}\;\text{sup}}\left\langle \left(A-\gamma f\right){℘}^{⋆},{℘}^{⋆}-{℘}_{q}\right\rangle \leq 0,\forall q\in N, \end{array}$$ (55)

where ${℘}^{⋆}=P_{F}\left(1-A+\gamma f\right){℘}^{⋆}$ represents a unique solution of the variational inequality (8). To start with, select a subsequence $\left\{{℘}_{q_{k_{\varsigma }}}\right\}$ of $\left\{{℘}_{q_{k}}\right\}$ such that

$$\begin{array}{c} \underset{\varsigma \to \infty }{\text{lim}}\left\langle \left(A-\gamma f\right){℘}^{⋆},{℘}^{⋆}-{℘}_{n_{k}}\right\rangle =\underset{q\to \infty }{\text{lim}}\left\langle \left(A-\gamma f\right){℘}^{⋆},{℘}^{⋆}-{℘}_{n}\right\rangle . \end{array}$$ (56)

Now, consequent upon the bounded of the sequence ${\left\{{℘}_{q}\right\}}_{n=0}^{\infty }$ (as shown above), we can find a subsequence $\left\{{℘}_{q_{k_{\varsigma }}}\right\}$ of $\left\{{℘}_{q_{k}}\right\}$ such that ${℘}_{q_{k}}⇀{\xi^{o}}^{⋆}\in F$ as *k* → ∞. Since ‖*u*_*q*_ − ℘_*q*_‖ → 0 *as* *q* → ∞, it follows that $u_{q_{k}⇀}{\xi^{o}}^{⋆}\;as\;k\to \infty$. We prove that ${\xi^{o}}^{⋆}\in F$.

To start with, we prove that *ξ*^*o*⋆^ ∈ *EP*(Ψ). By *u*_*q*_ = *Tr*_*q*_℘_*n*_, we get

$$\begin{array}{c} ℧\left(u_{q},y\right)+\frac{1}{r_{q}}\left\langle \hbar -u_{q},u_{q}-{℘}_{q}\right\rangle \geq 0,\;\forall \hbar \in Q. \end{array}$$

Using (B2), we also obtain

$$\begin{array}{c} \frac{1}{r_{q}}\left\langle \hbar -u_{q},u_{q}-{℘}_{q}\right\rangle \geq ℧\left(\hbar ,u_{q}\right),\;\forall \hbar \in Q, \end{array}$$

which consequently becomes,

$$\begin{array}{c} \left\langle \hbar -u_{q_{k}},\frac{u_{q_{k}}-{℘}_{q_{k}}}{r_{q_{k}}}\right\rangle \geq ℧\left(\hbar ,u_{n_{k}}\right). \end{array}$$

Since $\frac{u_{q_{k}}-{℘}_{q_{k}}}{r_{q_{k}}}\to 0$ and $u_{q_{k}}⇀{\xi^{o}}^{⋆}\;as\;k\to \infty$, from (B4), we obtain

$$\begin{array}{c} 0\geq ℧(\hbar ,{\xi^{o}}^{⋆}),\forall \hbar \in Q. \end{array}$$

Let ℏ_*t*_ = *t*ℏ + (1 − *t*)*ξ*^*o*⋆^, where ℏ ∈ *Q* and *t* ∈ (0, 1]. Since ℏ, *ξ*^*o*⋆^ ∈ *Q* and *Q* is convex, we get ℏ_*t*_ ∈ *Q* and ℧(*y*_*t*_, *ξ*^*o*⋆^) ≤ 0. Therefore, from (B1)and (B4), we get

$$\begin{array}{ccc} 0 & = & ℧(\hbar_{t},\hbar_{t})\\ & \leq & t℧\left(\hbar_{t},\hbar \right)+\left(1-t\right)℧\left(\hbar_{t},{\xi^{o}}^{⋆}\right)\\ & \leq & t℧(\hbar_{t},\hbar ), \end{array}$$

which yields ℧(ℏ_*t*_, ℏ) ≥ 0. Using (B3), we get ℧(*ξ*^*o*⋆^, ℏ) ≥ 0, ∀ℏ ∈ *Q*. Thus, *ξ*^*o*⋆^ ∈ *EP*(℧).

Now, from $u_{n_{k}}⇀{\xi^{o}}^{⋆}$, ${\text{lim}}_{q\to \infty }\left\|u_{q}-{ℑ}_{\varsigma }u_{q}\right\|=0$ and the demiclosedness property of ${ℑ}_{\varsigma }$ for each *ς*, and by applying standard argument, we have that ${\xi^{o}}^{⋆}\in \cap_{\varsigma =1}^{m}F\left({ℑ}_{\varsigma }\right)$. In addition, since ${\text{lim}}_{q\to \infty }\left\|\omega_{q}-{ð}_{\varsigma }\omega_{q}\right\|=0$ and lim_*q*→∞_ ‖*u*_*q*_ − *s*_*q*_‖ = 0, we immediately obtained from the demiclosedness property ${ð}_{\varsigma }$ that ${\xi^{o}}^{⋆}\in \cap_{\varsigma =1}^{m}F\left({ð}_{\varsigma }\right)$. Hence, ${\xi^{o}}^{⋆}\in F$. Since ${℘}^{⋆}=P_{F}\left(I-A+\gamma f\right){℘}^{⋆}$ and ${\xi^{o}}^{⋆}\in F$, we get from (55) that

$$\begin{array}{ccc} \underset{q\to \infty }{\text{lim}\;\text{sup}}\left\langle \left(A-\gamma f\right){℘}^{⋆},{℘}^{⋆}-{℘}_{q}\right\rangle & = & \underset{\varsigma \to \infty }{\text{lim}}\left\langle \left(A-\gamma f\right){℘}^{⋆},{℘}^{⋆}-{℘}_{n_{k_{i}}}\right\rangle \\ & = & \underset{\varsigma \to \infty }{\text{lim}}\left\langle \left(A-\gamma f\right){℘}^{⋆},{℘}^{⋆}-{\xi^{o}}^{⋆}\right\rangle \\ & \leq 0 \end{array}$$ (57)

as required.

Since from (23) and Lemma 2.2

$$\begin{array}{ccc} \|{℘}_{q+1}-{℘}^{⋆}{\|}^{2} & \leq & \|\alpha_{q}\gamma f\left({℘}_{q}\right)+\delta_{q}s_{q}+\left(\left(1-\delta_{q}\right)I-\alpha_{q}A\right)s_{q}-{℘}^{⋆}{\|}^{2}\\ & = & \|\alpha_{q}\left(\gamma f\left({℘}_{q}\right)-A{℘}^{⋆}\right)+\delta_{q}\left(s_{q}-{℘}^{⋆}\right)+\left(\left(1-\delta_{q}\right)I-\alpha_{q}A\right)\left(s_{q}-q\right){\|}^{2}\\ & \leq & \|\left(\left(1-\delta_{q}\right)-\alpha_{q}A\right)\left(s_{q}-{℘}^{⋆}\right)\\ & - & \delta_{q}\left({℘}^{⋆}-s_{q}\right){\|}^{2}+2\alpha_{q}\left\langle \gamma f\left({℘}_{q}\right)-A{℘}^{⋆},{℘}_{n+1}-q\right\rangle \\ & \leq & {\left(\left(1-\delta_{q}\right)I-\alpha_{q}\alpha \right)}^{2}\|s_{q}-{℘}^{⋆}{\|}^{2}+\delta_{q}^{2}\|s_{q}\\ & - & {℘}^{⋆}{\|}^{2}+2\alpha_{q}\gamma \left\langle f\left({℘}_{q}\right)-f\left({℘}^{⋆}\right),{℘}_{q+1}-{℘}^{⋆}\right\rangle \\ & & +2\alpha_{q}\left\langle \left(A-\gamma f\right){℘}^{⋆},{℘}^{⋆}-{℘}_{q+1}\right\rangle \\ & \leq & \left[1-2\delta_{q}+\delta_{q}^{2}-2\left(1-\delta_{q}\right)\alpha_{n}\alpha +\alpha_{q}^{2}\alpha^{2}\right]\|s_{q}-{℘}^{⋆}{\|}^{2}+\delta_{q}^{2}{\left\|s_{n}-{℘}^{⋆}\right\|}^{2}\\ & & +2\alpha_{q}\gamma \rho \|{℘}_{n}-℘\|\|{℘}_{q+1}-{℘}^{⋆}\|+2\alpha_{q}\left\langle \left(A-\gamma f\right){℘}^{⋆},{℘}^{⋆}-{℘}_{q+1}\right\rangle \\ & = & \left[1+2\delta_{q}\left(\delta_{n}-\alpha_{q}\alpha -1\right)-2\alpha_{q}\alpha +\alpha_{q}^{2}\alpha^{2}\right]\|s_{q}-{℘}^{⋆}{\|}^{2}+\alpha_{q}\gamma \rho [\|{℘}_{q}-{℘}^{⋆}{\|}^{2}\\ & & +\|{℘}_{q+1}-{℘}^{⋆}{\|}^{2}]+2\alpha_{q}\left\langle \left(A-\gamma f\right){℘}^{⋆},{℘}^{⋆}-{℘}_{q+1}\right\rangle \\ & \leq & \left(1-2\alpha_{q}\alpha \right)\|s_{q}-{℘}^{⋆}{\|}^{2}+\alpha_{q}^{2}\alpha^{2}\|s_{q}-{℘}^{⋆}{\|}^{2}+\alpha_{q}\gamma \rho [\|{℘}_{q}-{℘}^{⋆}{\|}^{2}\\ & & +\|{℘}_{q+1}-{℘}^{⋆}{\|}^{2}]+2\alpha_{q}\left\langle \left(A-\gamma f\right){℘}^{⋆},{℘}^{⋆}-{℘}_{q+1}\right\rangle , \end{array}$$ (58)

it follows from (25), (31) and (32) that

$$\begin{array}{ccc} \|{℘}_{q+1}-{℘}^{⋆}{\|}^{2} & \leq & \left(1-2\alpha_{q}\alpha \right)\|\omega_{q}-{℘}^{⋆}{\|}^{2}+\sum_{\xi^{o}=2}^{\upsilon }{\beta^{o}}_{q,\xi^{o}}\prod_{\tau =1}^{\xi^{o}-1}\left(1-{\beta^{o}}_{q,\tau }\right)\left(\nu_{q}^{2}-1\right){\left\|\omega_{q}-{℘}^{⋆}\right\|}^{2}\\ & & +\prod_{\tau =1}^{\upsilon }\left(1-{\beta^{o}}_{q,\tau }\right)\left(\nu_{q}^{2}-1\right)\|\omega_{q}-{℘}^{⋆}{\|}^{2}+\alpha_{q}^{2}\alpha^{2}{\left\|s_{q}-{℘}^{⋆}\right\|}^{2}\\ & & +\alpha_{q}\gamma \rho [\|{℘}_{q}-{℘}^{⋆}{\|}^{2}+\|{℘}_{q+1}-{℘}^{⋆}{\|}^{2}]+2\alpha_{q}\left\langle \left(A-\gamma f\right){℘}^{⋆},{℘}^{⋆}-{℘}_{q+1}\right\rangle \\ & \leq & \left(1-2\alpha_{q}\alpha \right)\|{℘}_{q}-{℘}^{⋆}{\|}^{2}+\sum_{\xi^{o}=2}^{\upsilon }\zeta_{q,\xi^{o}}\prod_{\tau =1}^{\xi^{o}-1}\left(1-\zeta_{q,\tau }\right)\left(\nu_{q}^{2}-1\right){\left\|u_{q}-{℘}^{⋆}\right\|}^{2}\\ & + & \prod_{\tau =1}^{\upsilon }\left(1-\zeta_{q,\tau }\right)\left(\nu_{q}^{2}-1\right){\left\|u_{q}-{℘}^{⋆}\right\|}^{2}\\ & + & \sum_{\xi^{o}=2}^{\upsilon }{\beta^{o}}_{q,\xi^{o}}\prod_{\tau =1}^{\xi^{o}-1}\left(1-{\beta^{o}}_{q,\tau }\right)\left(\nu_{q}^{2}-1\right){\left\|\omega_{q}-{℘}^{⋆}\right\|}^{2} \end{array}$$

$$\begin{array}{ccc} & & +\prod_{\tau =1}^{\upsilon }\left(1-{\beta^{o}}_{q,\tau }\right)\left(\nu_{q}^{2}-1\right)\|\omega_{q}-{℘}^{⋆}{\|}^{2}+\alpha_{q}^{2}\alpha^{2}\|s_{q}-{℘}^{⋆}{\|}^{2}+\alpha_{q}\gamma \rho [\|{℘}_{q}-{℘}^{⋆}{\|}^{2}\\ & & +\|{℘}_{q+1}-{℘}^{⋆}{\|}^{2}]+2\alpha_{q}\left\langle \left(A-\gamma f\right){℘}^{⋆},{℘}^{⋆}-{℘}_{q+1}\right\rangle \\ & \leq & \left[1-\left(2\bar{\alpha }-\gamma \rho \right)\alpha_{q}\right]{\left\|{℘}_{q}-{℘}^{⋆}\right\|}^{2}+\sum_{\xi^{o}=2}^{\upsilon }{\beta^{o}}_{q,\xi^{o}}\prod_{\tau =1}^{\xi^{o}-1}\left(1-{\beta^{o}}_{q,\tau }\right)\left(\nu_{q}^{2}-1\right)\varpi^{⋆}\\ & & +\prod_{\tau =1}^{\upsilon }\left(1-\zeta_{q,j}\right)\left(\nu_{q}^{2}-1\right)\varpi^{⋆}+\sum_{\xi^{o}=2}^{\upsilon }{\beta^{o}}_{q,\xi^{o}}\prod_{\tau =1}^{\xi^{o}-1}\left(1-{\beta^{o}}_{q,\tau }\right)\left(\nu_{q}^{2}-1\right)\varpi^{⋆⋆}\\ & & +\prod_{\tau =1}^{\upsilon }\left(1-{\beta^{o}}_{q,\tau }\right)\left(\nu_{q}^{2}-1\right)\varpi^{⋆⋆}+\alpha_{q}^{2}\alpha^{2}\varpi^{⋆⋆}+\alpha_{q}\gamma \rho {\left\|{℘}_{q+1}-{℘}^{⋆}\right\|}^{2}\\ & & +2\alpha_{q}\left\langle \left(A-\gamma f\right){℘}^{⋆},{℘}^{⋆}-{℘}_{q+1}\right\rangle \\ & \leq & \left[1-\left(2\bar{\alpha }-\gamma \rho \right)\alpha_{q}\right]{\left\|{℘}_{q}-{℘}^{⋆}\right\|}^{2}+\{\alpha_{q}^{2}\alpha^{2}+2\left(\nu_{q}^{2}-1\right)[\sum_{\xi^{o}=2}^{\upsilon }{\beta^{o}}_{q,\xi^{o}}\prod_{\tau =1}^{\xi^{o}-1}\left(1-\zeta_{q,\tau }\right)\\ & & +\prod_{\tau =1}^{\upsilon }\left(1-{\beta^{o}}_{q,\tau }\right)]\varpi \}+\alpha_{q}\gamma \rho {\left\|{℘}_{q+1}-{℘}^{⋆}\right\|}^{2}+2\alpha_{q}\left\langle \left(A-\gamma f\right){℘}^{⋆},{℘}^{⋆}-{℘}_{q+1}\right\rangle , \end{array}$$

where *ϖ*, *ϖ*^⋆^ and *ϖ*^⋆⋆^ are still as described above.

From the last inequality, we obtain that

$$\begin{array}{ccc} \|{℘}_{q+1}-{℘}^{⋆}{\|}^{2} & \leq & [1-\frac{2\left(\bar{\alpha }-\gamma \rho \right)\alpha_{n}}{1-\alpha_{q}\gamma \rho }]{\left\|{℘}_{q}-{℘}^{⋆}\right\|}^{2}+\frac{\alpha_{q}}{1-\alpha_{q}\gamma \rho }\{\{\alpha_{q}\alpha^{2}\\ & & +\frac{2(\nu_{q}^{2}-1)}{\alpha_{q}}[\sum_{\xi^{o}=2}^{\upsilon }{\beta^{o}}_{q,\xi^{o}}\prod_{\tau =1}^{\xi^{o}-1}\left(1-{\beta^{o}}_{q,\tau }\right)+\prod_{\tau =1}^{\upsilon }\left(1-{\beta^{o}}_{q,\tau }\right)]\}\varpi \\ & & +2\langle \left(A-\gamma f\right){℘}^{⋆},{℘}^{⋆}-{℘}_{q+1}\rangle \} \end{array}$$ (59)

Set

$$\begin{array}{ccc} \kappa_{q} & = & \frac{2\left(\bar{\alpha }-\gamma \rho \right)\alpha_{q}}{1-\alpha_{q}\gamma \rho },\\ \phi_{q} & = & \frac{\alpha_{q}}{1-\alpha_{q}\gamma \rho }\{\{\alpha_{q}\alpha^{2}+\frac{2(\nu_{q}^{2}-1)}{\alpha_{q}}[\sum_{\xi^{o}=2}^{\upsilon }{\beta^{o}}_{q,\xi^{o}}\prod_{\tau =1}^{\xi^{o}-1}\left(1-{\beta^{o}}_{q,\tau }\right)+\prod_{\tau =1}^{\upsilon }\left(1-{\beta^{o}}_{q,j}\right)]Q\}\\ & & +2\langle \left(A-\gamma f\right){℘}^{⋆},{℘}^{⋆}-{℘}_{q+1}\rangle \} \end{array}$$

and

$$\begin{array}{ccc} \psi_{q} & = & \frac{\phi_{q}}{\kappa_{q}}=\frac{1}{2(\bar{\alpha }-\gamma \rho )}\{\{\alpha_{q}\alpha^{2}+\frac{2(\nu_{q}^{2}-1)}{\alpha_{q}}[\sum_{\xi^{o}=2}^{\upsilon }{\beta^{o}}_{q,\xi^{o}}\prod_{\tau =1}^{\xi^{o}-1}\left(1-{\beta^{o}}_{q,\tau }\right)+\prod_{\tau 1}^{\upsilon }\left(1-{\beta^{o}}_{q,\tau }\right)]\varpi \}\\ & & +2\langle \left(A-\gamma f\right){℘}^{⋆},{℘}^{⋆}-{℘}_{q+1}\rangle \} \end{array}$$

Then, from (59), we have that

$$\begin{array}{c} b_{q+1}\leq \left(1-\kappa_{q}\right)b_{q}+\psi_{q}, \end{array}$$ (60)

where *b*_*q*_ = ‖℘_*q*_ − ℘^⋆^‖^2^. It is not difficult to see, from (iv) and the fact that $\underset{q\to \infty }{\text{lim}}\nu_{q}=1$, that

$$\begin{array}{c} \kappa_{q}\to 0\;as\;q\to \infty ,\sum_{q=1}^{\infty }\kappa_{q}=\infty \;\text{and}\;\underset{q\to \infty }{\text{lim}\;\text{sup}}\psi_{q}\leq 0. \end{array}$$

Thus, from Lemma 2.3 and (60), ${℘}_{q}\to {℘}^{⋆}\in F$.

Case B: Suppose {‖℘_*q*_ − *q*‖} is monotonically increasing. Then, the integer sequence ${\left\{\tau_{q}\right\}}_{q=0}^{\infty }$ (for some *q*_0_ large enough) can be written as

$$\begin{array}{c} \tau_{q}=max\{k\in N:k\leq q:\|{℘}_{k}-{℘}^{⋆}\left\|<\right\|{℘}_{k+1}-{℘}^{⋆}\left\|\right\}. \end{array}$$ (61)

It is easily seen that {*τ*_*q*_} is nondecreaing sequence and for all *q* ≥ *q*_0_, we have

$$\begin{array}{c} \|{℘}_{\tau (q)}-{℘}^{⋆}\left\|<\right\|{℘}_{\tau (q)+1}-{℘}^{⋆}\| \end{array}$$ (62)

From (40), (43), (48) and (43) with (*q* replaced by *τ*_(*q*)_), we obtain

$$\begin{array}{c} \underset{n\to \infty }{\text{lim}}\left\|u_{\tau (q)}-{ℑ}_{\varsigma }u_{\tau (q)}\right\|=0 \end{array}$$ (63)

and

$$\begin{array}{c} \underset{q\to \infty }{\text{lim}}\left\|{℘}_{\tau (q)}-u_{\tau (q)}\right\|=0 \end{array}$$ (64)

By using similar argument as in Case A, we have

$$\begin{array}{c} b_{\tau (q)+1}\leq \left(1-\nu_{\tau (q)}\right)b_{\tau (q)}+\psi_{\tau (q)}, \end{array}$$ (65)

*κ*_*τ*(*q*)_ → 0 as $\;q\to \infty ,\sum_{q=1}^{\infty }\kappa_{\tau (q)}=\infty$ and $\underset{q\to \infty }{\text{lim}\;\text{sup}}\psi_{\tau (q)}\leq 0$. Therefore, from Lemma 2.3, we obtain lim_*n*→∞_‖℘_*τ*(*q*)_ − ℘^⋆^‖ = 0 and $\underset{q\to \infty }{\text{lim}}\left\|{℘}_{\tau (q)+1}-{℘}^{⋆}\right\|=0$.

Hence, by Lemma 2.5, we get

$$\begin{array}{c} 0\leq \|{℘}_{q}-{℘}^{⋆}\left\|\leq max\{\right\|{℘}_{q}-{℘}^{⋆}\left\|,\right\|{℘}_{\tau (q)}-{℘}^{⋆}\left\|\right\}\underset{q\to \infty }{\text{lim}}\leq \left\|{℘}_{\tau (q)+1}-{℘}^{⋆}\right\|.t \end{array}$$

Hence, ${\left\{{℘}_{q}\right\}}_{n=0}^{\infty }$ converges to ${℘}^{⋆}=P_{F}\left(1-A+\gamma f\right){℘}^{⋆}$ and the proof is completed.

Next, using our main result (Theorem 2.1), we prove strong convergence theorem for finding a solution of the variational inequality problems in the setup of real Hilbert spaces.

**Theorem 3.2** *Let* $H,Q,{\left\{{ð}_{\varsigma }\right\}}_{\varsigma =1}^{N},{\left\{{ℑ}_{\varsigma }\right\}}_{\varsigma =1}^{N},F,A$ *and* *f*_*ς*_ *be as given in Theorem 3.1. Let* ${\left\{{℘}_{q}\right\}}_{n=0}^{\infty }$ *be a sequence developed from an arbitrary* ℘_0_ ∈ *Q* *by*

$$\begin{array}{c} \left\{ \begin{array}{cc} u_{q}∍\left\langle \hbar -u_{q},u_{q}-{℘}_{q}\right\rangle \geq 0,\forall \hbar \in Q; & \\ \omega_{q}=\gamma_{n,1}u_{q}+\sum_{\xi^{o}=2}^{\upsilon }\zeta_{q,\xi^{o}}\prod_{\tau =1}^{\xi^{o}-1}\left(1-\zeta_{q,\tau }\right)\vartheta_{q,\varsigma -1}+\prod_{\tau =1}^{\upsilon }\left(1-\zeta_{q,j}\right)\vartheta_{q,N}; & \\ s_{q}={\beta^{o}}_{q,1}\omega_{q}+\sum_{\xi^{o}=2}^{\upsilon }{\beta^{o}}_{q,\xi^{o}}\prod_{\tau =1}^{\xi^{o}-1}\left(1-{\beta^{o}}_{q,\tau }\right)\pi_{q,\varsigma -1}+\prod_{\tau =1}^{\upsilon }\left(1-{\beta^{o}}_{q,j}\right)\pi_{q,\upsilon }; & \\ {℘}_{q+1}=P_{Q}\left(\alpha_{q}\gamma f\left({℘}_{q}\right)+\delta_{q}s_{q}+\left(\left(1-\delta_{q}\right)I-\alpha_{q}A\right)s_{q}\right), \end{array}\right. \end{array}$$ (66)

*where* $\vartheta_{q,i}\in {ℑ}_{\xi^{o}}u_{q}$ *and* $\pi_{q,\xi^{o}}\in {ð}_{\xi^{o}}^{q}\omega_{q}$ *for each ξ*^*o*^, {*α*_*q*_}, {*δ*_*q*_} ∈ [0, 1] *and* ${\left\{{\left\{\zeta_{q,\xi^{o}}\right\}}_{q=1}^{\infty }\right\}}_{\xi^{o}=1}^{N},{\left\{{\left\{{\beta^{o}}_{q,\xi^{o}}\right\}}_{q=1}^{\infty }\right\}}_{\varsigma =1}^{N}\subseteq \left[0,1\right]$. *Suppose the requirements below are fulfilled*:

(*i*) ${\beta^{o}}_{q,1}\geq \zeta_{q,1}>max{\left\{k_{\xi^{o}}\right\}}_{\xi^{o}=1}^{N}:{\beta^{o}}_{q,\xi^{o}}\leq \zeta_{q,\xi^{o}}<\gamma \leq \beta^{o}<1,$ *for each* *ξ*^*o*^;
(*ii*) ${\text{lim}\;\text{inf}}_{q\to \infty }{\beta^{o}}_{q,\xi^{o}}\prod_{\tau =1}^{\upsilon }\left(1-{\beta^{o}}_{q,\tau }\right)\left({\beta^{o}}_{q,1}-k_{\varsigma -1}\right)>0$ *and* ${\text{lim}\;\text{inf}}_{q\to \infty }\prod_{\tau =1}^{\upsilon }\left(1-{\beta^{o}}_{q,j}\right)({\beta^{o}}_{q,1}-k_{N}>0;$
(*iii*) ${\text{lim}\;\text{inf}}_{n\to \infty }\zeta_{q,i}\prod_{j=1}^{\upsilon }\left(1-\zeta_{q,j}\right)\left(\zeta_{q,1}-k_{\varsigma -1}\right)>0$ *and* ${\text{lim}\;\text{inf}}_{q\to \infty }\prod_{\tau =1}^{\upsilon }\left(1-\zeta_{q,j}\right)(\gamma_{q,1}-k_{N}>0;$
(*iv*) ${\text{lim}}_{q\to \infty }\alpha_{q}=0,\sum_{q=1}^{\infty }\alpha_{q}=\infty$ *and* ${\text{lim}}_{q\to \infty }\frac{\left(1-{\beta^{o}}_{q,j}\right)\left(\nu_{q}^{2}-1\right)}{\alpha_{q}}=0$.

*Then*, ${℘}_{q}\to {℘}^{⋆}\in F$ *as q* → ∞, *which provides a solution to the variational inequality* $\langle \left(A-\gamma g\right){℘}^{⋆},℘-{℘}^{⋆}\rangle \geq 0,\forall ℘\in F$.

If ℧(℘, ℏ) = 0 ∀℘, ℏ∈*Q*, *r* = 1 ∀*q* ≥ 0, then *u*_*q*_ = ℘_*q*_. Therefore, with *f*(℘) = *v* and *A* = *I*, the conclusion is a consequence of Theorem 3.1.

## 4 Numerical example

Now, we present a numerical example to support to demonstrate the efficiency of our suggested method.

**Example 4.1** *Let Q* = [−3, 3] *and* H=R. *For each ξ*^*o*^ = 1, 2, 3, *let* ${ℑ}_{\xi^{o}}:q\to P\left(Q\right)$ *and* ${ð}_{\xi^{o}}:Q\to P\left(Q\right)$, *be given as*

$$\begin{array}{c} {ℑ}_{\xi^{o}}℘=[-{\left(\frac{5}{2}\right)}^{\xi^{o}}℘,-2^{\xi^{o}}℘] \end{array}$$ (67)

*and*

$$\begin{array}{c} {ð}_{xi}℘=[-\frac{1}{\sqrt{5^{\xi^{o}}}}℘,-\frac{1}{\sqrt{3^{\xi^{o}}}}℘]. \end{array}$$ (68)

*It is shown in* [11] *that* ℑ *is a k-SPM. Also, it is easy to see from Example 3.1 above that* ð *is k-SAPM. In addition, for n odd* (*q* ≥ 2), *we obtain*

$$\begin{array}{c} {ð}_{\xi^{o}}^{q}℘=[-\frac{1}{\sqrt{5^{\xi^{o}\left(2q-1\right)}}}℘,-\frac{1}{\sqrt{3^{\xi^{o}\left(2q-1\right)}}}℘]. \end{array}$$ (69)

*On the other hand, let the bifunction* ℧ *be given as*

$$\begin{array}{c} \left\{ \begin{array}{cc} ℧:Q\times Q⟶R & \\ ℧\left(℘,\hbar \right)=\hbar^{2}+℘\hbar -2{℘}^{2}. \end{array}\right. \end{array}$$ (70)

*It is easy to see that* ℧ *fulfills conditions* (*B*1) − (*B*4). *Set r*_*q*_ = *q* + 1, *then* $u_{q}=T_{r_{q}}\left({℘}_{q}\right)=\frac{{℘}_{q}}{3r_{q}+1}=\frac{{℘}_{q}}{3(q+1)+1}$, *where* *q* ≥ 1 (*see* [40] *for more information*). *For* *N* = 3, (23) *becomes*

$$\begin{array}{c} \left\{ \begin{array}{c} u_{q}∍℧\left(u_{q},y\right)+\frac{1}{r_{q}}\left\langle \hbar -u_{q},u_{q}-{℘}_{q}\right\rangle \geq 0,\forall \hbar \in Q\\ \omega_{q}=\zeta_{q1}u_{q}+\left(1-\zeta_{q,1}\right)\zeta_{q,2}\vartheta_{q,1}+\left(1-\zeta_{q,1}\right)\left(1-\zeta_{n,2}\right)\zeta_{q,3}\vartheta_{q,2}\\ +\left(1-\zeta_{q,1}\right)\left(1-\zeta_{q,2}\right)\left(1-\zeta_{q,3}\right)\vartheta_{n,3}\\ s_{q}={\beta^{o}}_{q,1}\omega_{q}+\left(1-{\beta^{o}}_{q,1}\right){\beta^{o}}_{q,2}\pi_{q,1}\\ +\left(1-{\beta^{o}}_{q,1}\right)\left(1-{\beta^{o}}_{q,2}\right){\beta^{o}}_{q,3}\pi_{q,2}+\left(1-{\beta^{o}}_{q,1}\right)\left(1-{\beta^{o}}_{q,2}\right)\left(1-{\beta^{o}}_{q,3}\right)\pi_{q,3}\\ {℘}_{q+1}=P_{Q}\left(\alpha_{q}\gamma f\left({℘}_{q}\right)+\delta_{q}s_{q}+\left(\left(1-\delta_{q}\right)I-\alpha_{q}A\right)\right). \end{array}\right. \end{array}$$

*Put* $f\left(℘\right)=\frac{℘}{2},A=1,\zeta =1,\zeta_{q,1}=\zeta_{q,2}=\zeta_{q,3}=1-\frac{1}{q}\;\left(q\geq 2\right),{\beta^{o}}_{q,1}={\beta^{o}}_{q,2}={\beta^{o}}_{q,3}=\frac{1}{3}$ *and* $\alpha_{q}=\frac{1}{100q}$. *Then, for arbitrary* ℘_0_ ∈ *Q*, *the above iteration scheme yields*:

$$\begin{array}{c} \left\{ \begin{array}{cc} \omega_{q}=\frac{n-1}{(3r_{q}+1)n}{℘}_{q}+\frac{1}{q}(\frac{q-1}{q})\vartheta_{q,1}+\frac{1}{q^{2}}(\frac{q-1}{n})\vartheta_{q,2}+\frac{1}{q^{3}}\vartheta_{q,3} & \\ s_{q}=\frac{\omega_{q}}{3}+\frac{2}{9}\pi_{q,1}+\frac{2}{27}\pi_{q,2}+\frac{1}{27}\pi_{q.3} & \\ {℘}_{q+1}=\frac{1}{200q}{℘}_{q}+(1-\frac{1}{100q})s_{q}, \end{array}\right. \end{array}$$ (71)

*where* $\vartheta_{q,1}\in [-\frac{5}{2}{℘}_{q},-2{℘}_{q}],\vartheta_{q,2}\in [-\frac{25}{4}{℘}_{q},-4{℘}_{q}],\vartheta_{q,3}\in [-\frac{125}{8}{℘}_{q},-8{℘}_{q}]$ *for* ℘_*q*_ ∈ (−3, 0] *whereas* $\pi_{q,1}\in [-\frac{1}{\sqrt{3^{2q-1}}}{℘}_{n},-\frac{1}{\sqrt{5^{2q-1}}}{℘}_{n}],\pi_{q,2}\in [-\frac{1}{\sqrt{9^{2q-1}}}{℘}_{q},-\frac{1}{\sqrt{25^{2q-1}}}{℘}_{n}],\pi_{q,3}\in [-\frac{1}{\sqrt{27^{2q-1}}}{℘}_{q},-\frac{1}{\sqrt{125^{2q-1}}}{℘}_{q}]$ *if* ℘_*q*_ ∈ (−3, 0].

*Observe that the sequence* ℘_*q*_ → 0 *as q* → ∞. *To be precise*, $F=(\cap_{\xi^{o}=1}^{3}\left(F\left({ℑ}_{\xi^{o}}\right)\right)\cap \left(\cap_{\xi^{o}=1}^{3}F\left({ð}_{\xi^{o}}\right)\right)\cap EP\left(℧\right)=\left\{0\right\}$.

## 5 Conclusion

In this manuscript, we introduce a new class of mappings (*θ*-SAPM) and propose a novel method for solving equilibrium problem with mixed fixed point constraints. We establish strong convergence result of the proposed technique without any imposition of sum conditions on the iteration parameters (hence less computational cost). In addition, we showed that the class of *θ*-SPM and the class of *θ*-SAPM are independent. Also, we illustrated the convergence of our method through numerical experiment. Our future project will consider some comparison test of our technique with some existing techniques that probably imposes sum conditions on the iteration parameters.
